# MESH1-mediated coenzyme A degradation drives ferroptosis sensitivity and muscle pathology

**DOI:** 10.1172/JCI202212

**Published:** 2026-04-25

**Authors:** Chao-Chieh Lin, Joshua Rose, Alexander A. Mestre, Chien-Kung Cornelia Ding, Ssu-Yu Chen, Sze Mun Choy, Kah Yong Goh, Weiyi Jiang, Wen Xing Lee, Qizhou Jiang, Yanting Chen, Tianai Sun, Jianli Wu, Yueqi Chen, Yunju Oh, Pyeonghwa Jeong, Jiyong Hong, Kenon Chua, Michael C. Fitzgerald, Guo-Fang Zhang, Hong-Wen Tang, Pei Zhou, Jen-Tsan Chi

**Affiliations:** 1 Department of Molecular Genetics and Microbiology,; 2 Duke Center for Genomic and Computational Biology, and; 3 Department of Biochemistry, Duke University School of Medicine, Durham, North Carolina, USA.; 4 Program in Cancer and Stem Cell Biology, Duke-NUS Medical School, Singapore.; 5 Department of Anesthesiology, Duke University School of Medicine, Durham, North Carolina, USA.; 6 Department of Chemistry, Duke University, Durham, North Carolina, USA.; 7 Department of Pharmacology and Cancer Biology, Duke University School of Medicine, Durham, North Carolina, USA.; 8 Department of Orthopaedic Surgery, Singapore General Hospital, Singapore.; 9 Duke Molecular Physiology Institute and Sarah W. Stedman Nutrition and Metabolism Center, Duke University, Durham, North Carolina, USA.; 10 Department of Medicine, Division of Endocrinology, Metabolism, and Nutrition, Duke University School of Medicine, Durham, North Carolina, USA.

**Keywords:** Cell biology, Muscle biology, Amino acid metabolism, Molecular biology, Muscle

## Abstract

CoA facilitates fatty acid synthesis, energy production, gene regulation, and antioxidant function. While CoA biosynthesis is well characterized, the mechanisms governing CoA degradation remain poorly understood. Here, we identify the Metazoan Homolog of SpoT, MESH1, as a CoA phosphatase that dephosphorylates CoA at the 3′ position of the ribose ring to form dephospho-CoA. Recent studies have shown that CoA, similar to glutathione, is a cysteine-derived metabolite that protects cells against ferroptosis. Ferroptosis induced by blocking cystine import depletes CoA biosynthesis, while CoA restoration rescues cells from ferroptosis. We found that *MESH1* knockdown preserved CoA levels by preventing its degradation, contributing to ferroptosis protection, indicating the bifunctional role of MESH1 in regulating CoA and previously reported NADPH. Mechanistically, *MESH1* knockdown elevates CoA levels, maintaining a functional mitochondrial thioredoxin system, thereby preventing mitochondrial lipid peroxidation. In *Drosophila*, we found that *dMesh1* overexpression leads to ferroptosis-mediated muscle atrophy, which can be rescued by increasing CoA and NADPH levels. Taken together, these findings establish MESH1 as a key phosphatase that governs ferroptosis sensitivity by coordinating CoA and NADPH homeostasis, unveiling a link between CoA degradation, mitochondrial integrity, and muscle health.

## Abstract 2

MESH1 breaks down a key molecule (CoA), increasing vulnerability to oxidative damage and driving muscle loss, revealing new targets to prevent disease-related tissue damage.

## Graphical abstract

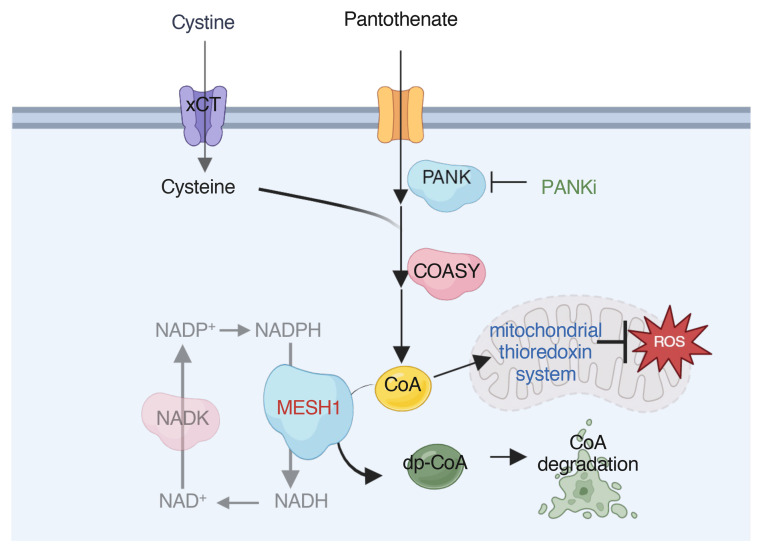

## Introduction

Ferroptosis was first identified as the mechanism through which erastin induces cell death by inhibiting the cystine/glutamate antiporter xCT, a transporter critical for importing cystine required for glutathione (GSH) synthesis (1). GSH serves as a cofactor for glutathione peroxidase 4 (GPX4) to reduce lipid hydroperoxides and prevent membrane rupture and ferroptosis (2). In addition to GSH synthesis, cysteine is also an intermediate metabolite for CoA biosynthesis (3). As a result, various xCT inhibitors can lead to CoA depletion and contribute to ferroptosis (3). CoA was first identified as a ferroptosis inhibitor in p53-mutant cells and was proposed to protect pancreatic tumors from ferroptosis by producing coenzyme Q10 to activate FSP1 (3, 4). We recently showed that CoA regulates the redox status of the mitochondrial thioredoxin system by covalently modifying Cys-483 of mitochondrial thioredoxin reductase (TXNRD2) via CoAlation (covalent modification by CoA) and promoting its enzymatic activity (5). Therefore, CoA depletion accelerates ferroptosis progression by impairing mitochondrial redox defense.

CoA biosynthesis begins with pantothenate kinase (PANK) phosphorylating extracellular pantothenate (vitamin B_5_) to form phosphopantothenate. This intermediate then reacts with cysteine, imported by xCT, to produce 4′-phosphopantothenoylcysteine, which is subsequently decarboxylated to 4′-phosphopantetheine. CoA synthase (COASY) then catalyzes a 2-step reaction: first, the addition of AMP to 4′-phosphopantetheine to form dephospho-CoA (dp-CoA) and, second, the phosphorylation of dp-CoA at the 3′-hydroxyl group by ATP, yielding CoA (6, 7). During CoA degradation, the first step is the dephosphorylation of CoA at the 3′ position of the ribose ring, which leads to dp-CoA (8). While this CoA phosphatase activity was detected a long time ago in several fractions from a tissue fractionation study (9), the identity of this enzyme has remained unclear.

All living organisms constantly face stressful conditions. In bacteria, the main strategy to cope with the metabolic stress is a “stringent response” triggered by the accumulation of the bacterial alarmone (p)ppGpp (10). (p)ppGpp levels are regulated by its synthetase RelA and hydrolase SpoT (RSH) (10). While highly conserved in bacteria and plants, no stringent response had been reported in metazoans until a study identified *Drosophila* and human MESH1 (encoded by *HDDC3*) as homologs of the bacterial SpoT (11) with similar (p)ppGpp hydrolase activity. However, the relevant substrates and functions of MESH1 were poorly defined. We first identified MESH1 in a ferroptosis screen, where its depletion conferred ferroptosis resistance (12). Furthermore, MESH1 removal triggered integrative stress (13) and reduced cellular proliferation (14) that mimicked many features of bacterial stringent response (15, 16). Mechanistically, we have shown that MESH1 is the first identified human NADPH phosphatase that contributes to NADPH depletion during ferroptosis (12). Notably, the NADPH phosphatase activities of MESH1 have been validated in bacteria (17) and *Caenorhabditis* *elegans* (18), suggesting that this enzymatic activity is evolutionarily conserved across multiple organisms.

CoA shares structural similarities with NADPH (12) and (p)ppGpp (11), 2 known MESH1 substrates that contain both ribose rings and adenine. Here, we identified MESH1 as a potent CoA phosphatase that catalyzes the dephosphorylation of CoA, driving its degradation. Beyond its previously established role in regulating NADPH, MESH1 also modulates CoA availability to maintain a functional mitochondrial thioredoxin system, thereby preventing ferroptosis. In *Drosophila*, *dMesh1* overexpression induced ferroptosis-associated muscle atrophy, driven by dual dephosphorylation of CoA and NADPH. Our findings demonstrate that MESH1 is a key CoA-degrading phosphatase that modulates CoA levels to regulate ferroptosis, revealing a link between CoA degradation, mitochondrial integrity, and ferroptosis sensitivity.

## Results

### MESH1 is an efficient CoA phosphatase.

The alarmone (p)ppGpp is regulated by SpoT/RSH enzymes in bacteria (10). Although (p)ppGpp appears absent or occurs at very low levels in metazoans (19, 20), the evolutionary conservation between bacterial SpoT and human MESH1 enables MESH1 to hydrolyze (p)ppGpp (11). Our previous research revealed that the enzymatic activity of MESH1 in humans can dephosphorylate NADPH to NADH (12), a result that has been independently validated in *C*. *elegans* (18) and bacteria (17). Given MESH1’s ability to hydrolyze NADPH — a metabolite structurally distinct from its bacterial ancestor’s substrate, (p)ppGpp — we hypothesized that MESH1 may exhibit broader substrate selectivity. Specifically, we explored whether MESH1 could act on other metabolites sharing similar nucleotide-based architectures. CoA emerged as a candidate due to its structural resemblance to (p)ppGpp (featuring a 3′ pyrophosphate) and NADPH (containing an adenosine nucleotide) (Supplemental Figure 1A; supplemental material available online with this article; https://doi.org/10.1172/JCI202212DS17).

**Figure 1:**
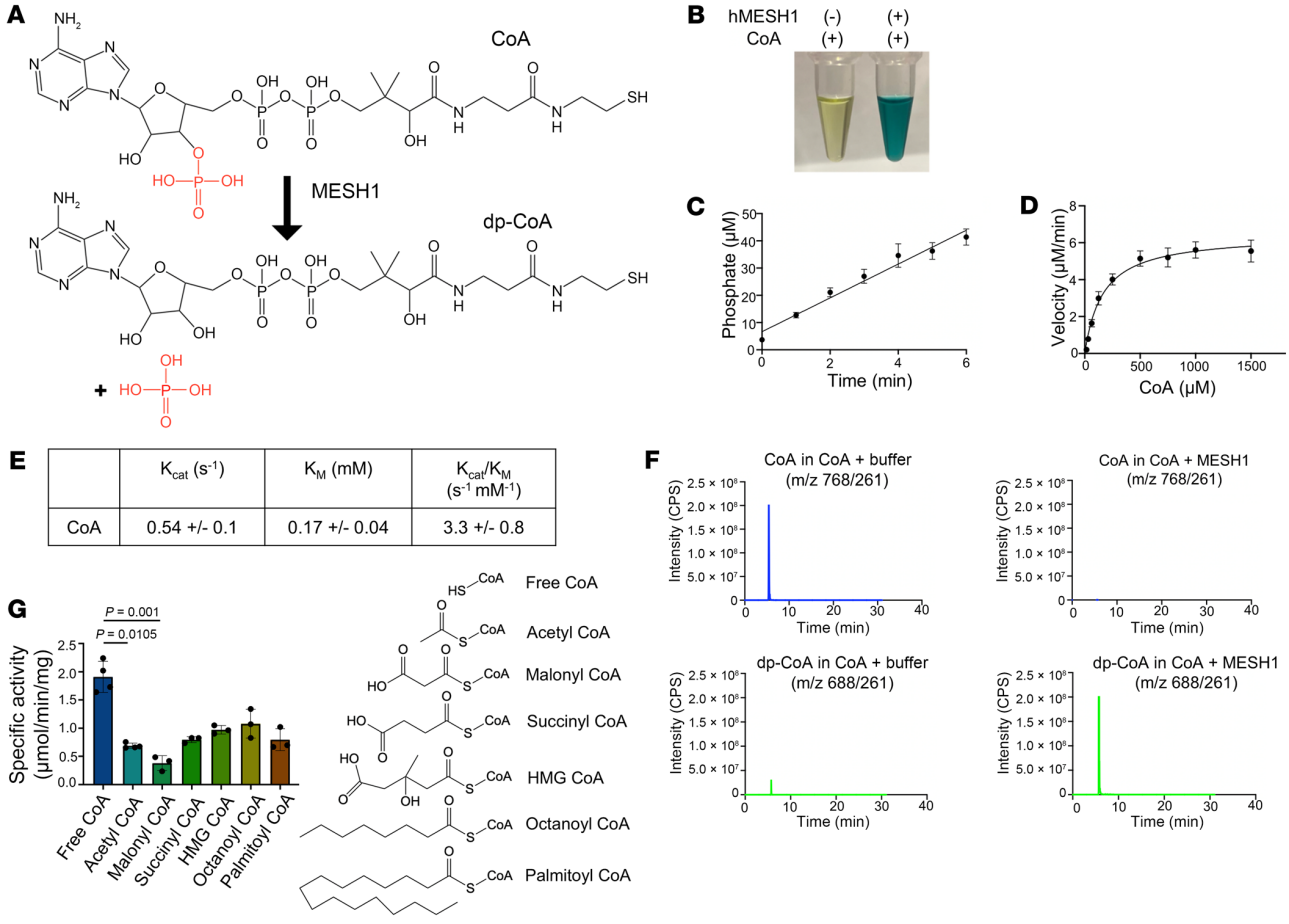
MESH1 is an efficient CoA phosphatase. (**A**) Proposed chemical reaction of MESH1 dephosphorylating the 3′ phosphate of CoA. (**B**) Detection of MESH1-dependent release of free phosphate from CoA using malachite green assay (phosphate accumulation causes yellow-to-green color transition). (**C**) Linear accumulation of phosphate product generated during MESH1 CoA reaction (*n* = 4 independent experiments; data are shown as mean ± SEM). (**D**) Fitting of steady-state Michaelis-Menten kinetics equation toward MESH1 CoA enzymatic reaction (*n* = 4 independent experiments, data are shown as mean ± SEM). (**E**) Kinetic properties of reaction (*n* = 4 independent experiments; data are shown as mean ± SD). (**F**) LC-MS/MS analysis of CoA/dp-CoA in the presence or absence of MESH1. (**G**) Measurement of specific activity of different CoA species, with the chemical structures of the thioester groups shown (*n* = 3 independent experiments; data are shown as mean ± SD). One-way ANOVA; nonparametric analysis (Kruskal-Wallis).

Therefore, we speculated that MESH1 could also hydrolyze CoA to form dp-CoA and inorganic phosphate (Figure 1A). To test this possibility, we incubated CoA with purified MESH1 protein and were able to detect a rapid release of inorganic phosphate using the colorimetric malachite green assay (Figure 1B). The MESH1 CoA reaction showed linear product accumulation over time (Figure 1C), and the steady state kinetics revealed that MESH1 hydrolyzes CoA with a catalytic efficiency (*k*_cat_/*K_m_*) of 3.3 ± 0.8 s^–1^·mM^–1^ (Figure 1, D and E). Although MESH1’s catalytic efficiency toward CoA is lower than MESH1’s activity toward NADPH (*k*_cat_/*K_m_*: 14.4 ± 1.1 s^–1^·mM^–1^) and ppGpp (*k*_cat_/*K_m_*: 9.46 s^–1^·mM^–1^), it still demonstrates substantial CoA phosphatase activity (Supplemental Figure 1B). The *K_m_* value of MESH1 for free CoA was calculated to be 170 ± 40 μM, comparable to the *K_m_* values of intracellular NUDIX enzymes, NUDT7 (240 μM), NUDT8 (150 ± 13 μM), and NUDT19 (300 ± 40 μM), which hydrolyze CoA to form 3′,5′-ADP and phosphopantetheine (21–23). Using liquid chromatography–tandem mass spectrometry (LC-MS/MS), we confirmed that incubation of CoA with MESH1, but not buffer alone, led to the complete conversion of CoA to dephosphorylated CoA (Figure 1F). These experiments support the notion that MESH1 functions as a CoA phosphatase.

Next, we assessed the ability of MESH1 to dephosphorylate a panel of CoA derivatives, including free CoA, acetyl-CoA, malonyl-CoA, succinyl-CoA, HMG-CoA, octanoyl-CoA, and palmitoyl-CoA (Figure 1G). We found that MESH1 can dephosphorylate all CoA species with the highest activity toward free CoA (1.8 ± 0.2 μmol/min/mg) and the least activity toward malonyl-CoA (0.38 ± 0.13 μmol/min/mg). The total cytosolic concentration of CoA species in liver cells has been estimated to range from 106 to 140 μM, with the free CoA making up 50–90 μM, acetyl-CoA 10–40 μM, and long-chain acyl-CoAs 30 μM (24). Given both the relatively high catalytic efficiency of MESH1 for free CoA and its intracellular abundance, free CoA is likely the physiologically most relevant CoA species impacted by MESH1 hydrolysis. These findings support CoA as an additional MESH1 substrate, expanding its role beyond NADPH regulation to CoA metabolism and cellular redox homeostasis.

### MESH1 knockdown preserves CoA levels to protect against ferroptosis.

**Figure 2:**
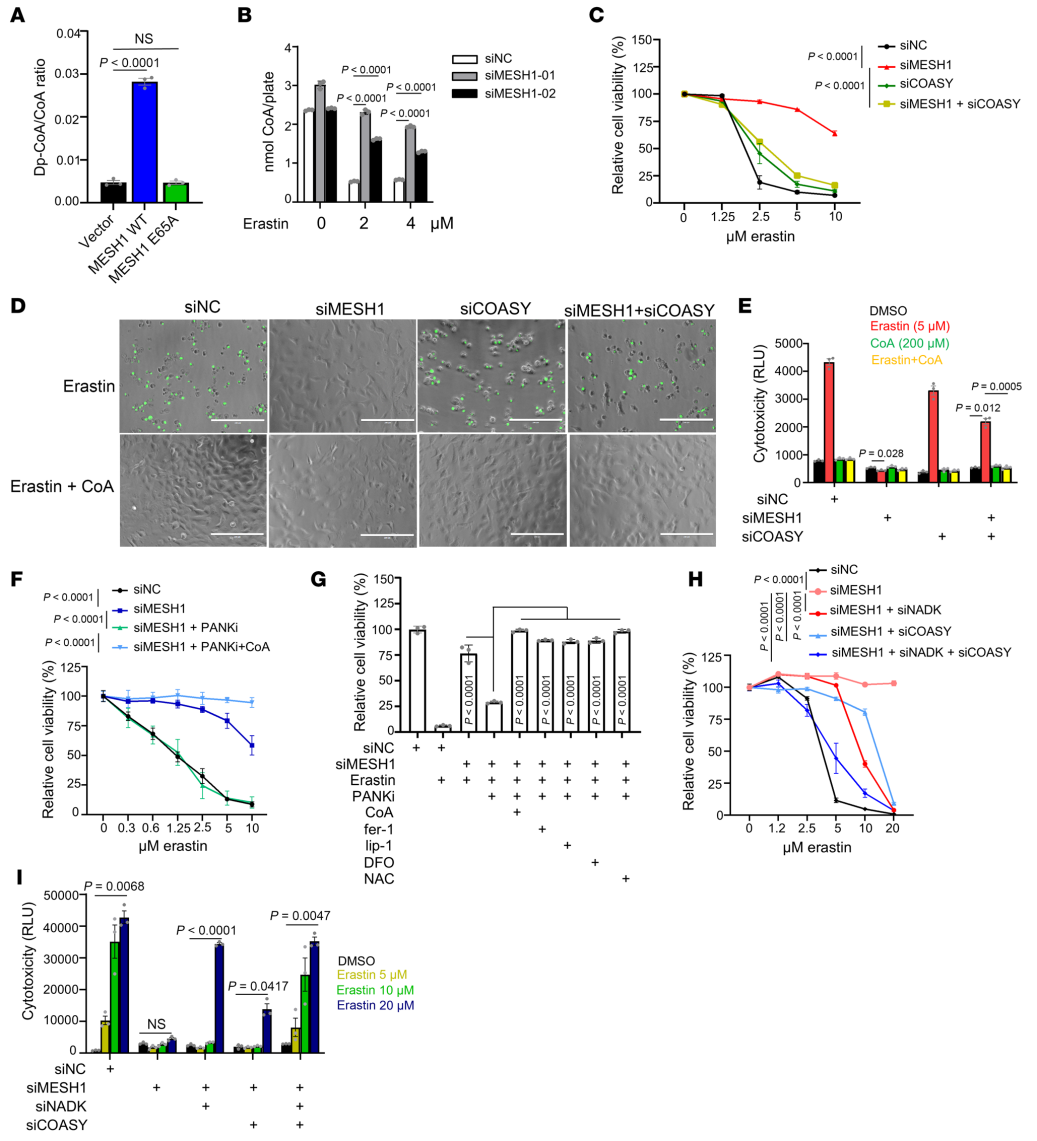
The CoA level preserved by *MESH1* knockdown protects against ferroptosis. (**A**) The dp-CoA/CoA ratio in HEK293T cells overexpressing empty vector, WT-MESH1, and the catalytically null mutant (MESH1-E65A) as determined by mass spectrometry. (**B**) *MESH1* knockdown preserved CoA levels degraded upon erastin treatment, as quantified by CoA assay. HT-1080 cells were transfected with control or 2 independent MESH1 siRNAs for 2 days and treated with erastin for 18 hours to quantify CoA level. (**C**) CoA biosynthesis is required for the protective effect of *MESH1* knockdown upon erastin treatment. HT-1080 cells were transfected individually or in combination with MESH1 and COASY siRNAs for 2 days, subjected to 24 hours of erastin treatment, and quantified by Cell-Titer Glo assay. (**D** and **E**) COASY knockdown abolished the protective effect of *MESH1* knockdown on erastin-induced (2.5 μM, 20 hours) membrane rupture in HT-1080 cells, while CoA supplement (100 μM) rescued membrane rupture in all conditions. The results were observed by CellTox Green under a fluorescence microscope (**D**) and quantified by a plate reader (**E**). Scale bars: 200 μm. (**F**) PANKi (5 μM, chemical inhibitor of PANK) abolished the protective effects of *MESH1* knockdown upon erastin treatment in HT-1080 cells. (**G**) PANKi (5 μM) abolished the protective effects of *MESH1* knockdown in HT-1080 cells by ferroptosis, and this resensitization was rescued by ferroptosis inhibitors. CoA (100 μM); ferrostatin-1 (fer-1, 10 μM); liproxstatin-1 (lip-1, 2 μM); deferoxamine (DFO, 80 μM); NAC (500 μM). (**H** and **I**) Double knockdown of NADK (NADPH synthesis) and COASY (CoA synthesis) fully abolished the protective effects of *MESH1* knockdown, as quantified by Cell-Titer Glo assay (**H**) and CellTox Green (**I**). (**A** and **G**) One-way ANOVA, Tukey’s multiple comparisons, *n* = 3 independent biological replicates. (**B**, **C**, **E**, **F**, **H**, and **I**) Two-way ANOVA, Šidák’s multiple comparisons, *n* = 3 independent biological replicates; data are shown as mean ± SEM.

To examine MESH1 enzymatic activity in regulating the dp-CoA/CoA ratio, we stably overexpressed an empty vector, WT-MESH1, and the catalytically inactive mutant MESH1-E65A (12) in HEK-293T cells (Supplemental Figure 2A). We found that WT- MESH1, but not the catalytically inactive mutant (MESH1-E65A), significantly increased the dp-CoA/CoA ratio, as determined by mass spectrometry, suggesting that MESH1 enzymatic activity catalyzes CoA dephosphorylation and degradation in HEK293T cells (Figure 2A).

Ferroptosis has been shown to reduce intracellular CoA levels by impairing cystine uptake and limiting CoA biosynthesis (3–5). To assess whether MESH1 contributes to CoA degradation during ferroptosis, we knocked down MESH1 expression using 2 independent siRNAs (Supplemental Figure 2B). While *MESH1* knockdown alone did not consistently increase CoA levels (Figure 2B), it significantly preserved CoA levels following erastin treatment (Figure 2B). Therefore, MESH1 may regulate CoA levels during ferroptosis.

Although *MESH1* knockdown did not expand the total CoA pool under basal conditions, we found that *MESH1* knockdown altered the metabolic function of xCT antiporter (encoded by *SLC7A11*), as evidenced by increased cystine and glutamate exchange activity reflected by enhanced glutamate export (Supplemental Figure 2C). Together, these data suggest that reduced MESH1-mediated CoA degradation becomes functionally important when CoA biosynthesis is limited during ferroptosis, rather than under basal conditions.

Ferroptosis can be induced by 4 classes of ferroptosis inducers (FINs) with class I targeting xCT transporter, class II targeting GPX4, class III depleting GPX4 and CoQ_10_, and class IV inducing lipid peroxidation (25). Given that cysteine is also required for CoA biosynthesis, consistent with our previous results (5), we found that *MESH1* knockdown or CoA supplementation exclusively protects against ferroptosis induced by class I inducers (Supplemental Figure 2D). To extend these observations to additional cellular contexts, we tested the protective effects of *MESH1* knockdown or CoA supplementation in *GPX4*-knockout HT-1080 cells (26) (Supplemental Figure 2E). Given that *GPX4* knockout triggers ferroptosis resembling class II FINs, we found that *MESH1* knockdown and CoA supplementation were unable to rescue *GPX4* knockout–induced ferroptosis (Supplemental Figure 2E). Consistently, we confirmed that ferrostatin-1 and liproxstatin-1, 2 radical-trapping antioxidants, effectively rescued *GPX4* knockout–induced ferroptosis, but not *N*-acetylcysteine (NAC), which replenishes the intracellular cysteine pool (Supplemental Figure 2E). Class II, III, and IV FINs generally act downstream of and independently from cystine import (27); therefore, these results suggest that intracellular cysteine levels and CoA biosynthesis may contribute substantially to the protective role of *MESH1* knockdown in class I FIN-induced ferroptosis.

To test whether CoA preservation contributed to the ferroptosis protection conferred by *MESH1* knockdown, we next performed concurrent knockdown of *COASY*, the final 2 steps in CoA biosynthesis (Supplemental Figure 2, F–H). As expected, *MESH1* knockdown protected HT-1080, RCC4, and MDA-MB-231 cells from erastin-induced ferroptosis, as measured by cell viability (Figure 2C and Supplemental Figure 2, I and J). Importantly, the protective effect of *MESH1* knockdown was abolished by concurrent *COASY* knockdown (Figure 2C and Supplemental Figure 2, I and J), indicating that CoA synthesis is required for the ferroptosis resistance conferred by *MESH1* depletion. To determine whether restoring CoA could reverse this effect, we supplemented the culture media with CoA, which is hydrolyzed extracellularly to 4′-phosphopantetheine to cross the membrane and increase intracellular CoA levels (3–5, 28). Indeed, CoA supplementation rescued the ferroptosis-sensitizing effect caused by *COASY* knockdown, as visualized by fluorescence microscopy with CellTox Green (Figure 2D) and quantified by cytotoxicity assay (Figure 2E).

To further confirm the requirement of CoA biosynthesis in MESH1-mediated ferroptosis protection, we treated cells with PANK inhibitor (PANKi), which blocks the first step of CoA biosynthesis from vitamin B_5_ (29). Similar to the *COASY* knockdown, PANKi treatment abolished the protective effect of *MESH1* knockdown against ferroptosis in both HT-1080 and RCC4 cells (Figure 2F and Supplemental Figure 2K), and this effect was also reversed by CoA supplementation (Figure 2F and Supplemental Figure 2K). To confirm that the resensitization by PANKi occurred through ferroptosis, we treated cells with several canonical ferroptosis inhibitors, including ferrostatin-1, liproxstatin-1, deferoxamine, and NAC (Figure 2G and Supplemental Figure 2L). All of these ferroptosis inhibitors fully rescued PANKi-sensitized cell death in both HT-1080 (Figure 2G) and RCC4 cells (Supplemental Figure 2L). Together, these results indicate that *MESH1* knockdown preserves intracellular CoA levels from de novo biosynthesis, thereby protecting cells against ferroptosis.

Our previous finding showed that MESH1 is an NADPH phosphatase (12), and *MESH1* knockdown protected against ferroptosis by preserving NADPH (12). Furthermore, the ferroptosis protection of *MESH1* knockdown is attenuated by the simultaneous knockdown of cytosolic NAD kinase (*NADK*), the enzyme responsible for the NADP^+^ synthesis from NAD^+^ (12). Thus, we validated this observation in MDA-MB-231 and A549 cells using both cell viability and cytotoxicity assays (Supplemental Figure 2, M–P). To complement this genetic approach, we supplemented the culture medium with nicotinamide (NAM), a cell-permeable NAD^+^ precursor known to enhance intracellular NADPH levels (28). NAM treatment further potentiated the protective effect of *MESH1* knockdown in 786-O and HK-2 cells (Supplemental Figure 2, Q and R). Conversely, NAM supplementation attenuated the ferroptosis-promoting effect of MESH1 overexpression (Supplemental Figure 2S). Together, these results demonstrate that NADPH availability is a critical determinant of MESH1-mediated ferroptosis regulation and support a functional role for the NADPH axis in the protective effect conferred by *MESH1* knockdown.

Building on our new findings that CoA is also a direct substrate of MESH1, we hypothesize that MESH1 could simultaneously regulate the metabolism of both CoA and NADPH, 2 key metabolites implicated in ferroptosis protection. To test this hypothesis, we performed combinatorial knockdown experiments using siRNAs targeting *NADK*, which is responsible for NADP^+^ synthesis, and COASY, which is responsible for CoA biosynthesis. Each knockdown condition was validated for efficacy (Supplemental Figure 2, T and U). We found that individual knockdown of either *NADK* or *COASY* partially reversed the ferroptosis resistance conferred by *MESH1* knockdown, as assessed by cell viability in HT-1080 and RCC4 cells (Figure 2H and Supplemental Figure 2V), cytotoxicity assays (Figure 2I), and lipid peroxidation measurement in MDA-MB-231 and HEK293 cells (Supplemental Figure 2, W–Y). Notably, simultaneous knockdown of both *NADK* and *COASY* completely abolished this protective effect (Figure 2, H and I, and Supplemental Figure 2, V–Y). These results support the conclusion that MESH1 regulates ferroptosis by coordinating the metabolism of CoA and NADPH.

### MESH1 knockdown preserves the mitochondrial thioredoxin system to protect against ferroptosis.

**Figure 3:**
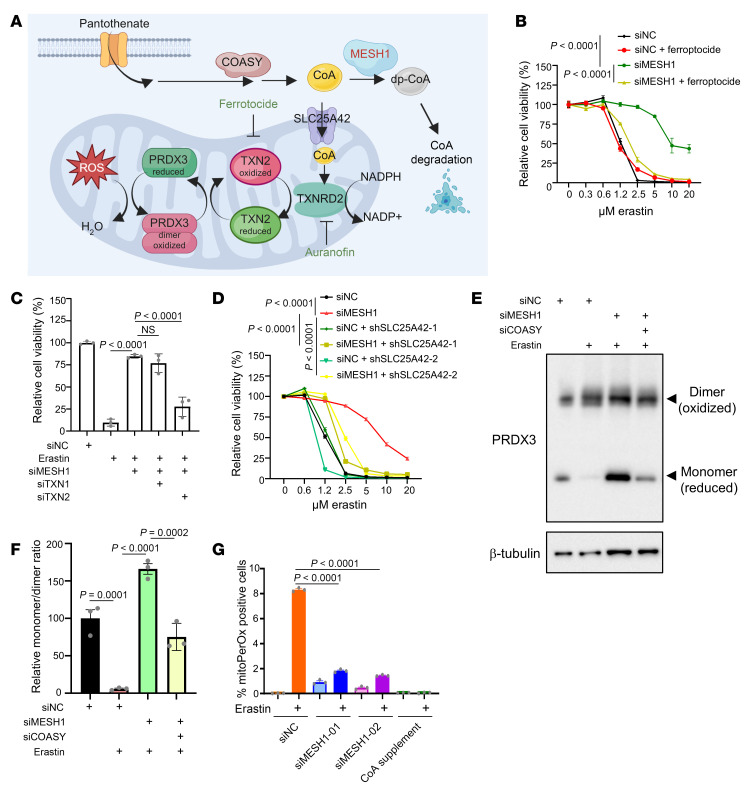
*MESH1* knockdown maintains functional mitochondrial thioredoxin system upon erastin treatment. (**A**) Graphical abstract of CoA import in maintaining the mitochondrial thioredoxin system. (**B**) Ferroptocide (2 μM, a chemical inhibitor of thioredoxin) abolished the protective effects of *MESH1* knockdown upon erastin treatment. HT-1080 cells were transfected with control or MESH1 siRNA for 2 days, treated with erastin for 18 hours, and quantified by Cell-Titer Glo assay. (**C**) Mitochondrial thioredoxin is required for the protective effect of *MESH1* knockdown upon erastin treatment. HT-1080 cells were transfected with MESH1 siRNA or in combination with cytosolic thioredoxin (TXN1) or mitochondrial thioredoxin (TXN2) for 2 days, subjected to 24 hours of erastin treatment, and quantified by Cell-Titer Glo assay. (**D**) Mitochondrial CoA transporter (SLC25A42) is required for the protective effect of *MESH1* knockdown. HT-1080 cells transduced with control or 2 independent SLC25A42 shRNAs were knocked down with MESH1 siRNA and erastin treatment for Cell-Titer Glo assay. (**E**) The lowering monomer/dimer ratio of PRDX3 upon erastin treatment was rescued by *MESH1* knockdown. Further knocking down of COASY abolished the protective effect of *MESH1* knockdown, as determined by Western blots. (**F**) Quantification of the monomer/dimer ratio of PRDX3 upon erastin treatment with or without MESH1 siRNA or in combination with COASY siRNA. (**G**) *MESH1* knockdown lowered erastin-induced mitochondrial lipid peroxidation. HT-1080 cells knocked down by control or 2 independent MESH1 siRNAs were treated with erastin (2 μM, 20 hours) or supplemented with CoA and quantified by the mitochondrial lipid peroxidation (mitoPerOx) sensor. (**C**, **F**, and **G**) One-way ANOVA, Tukey’s multiple comparisons, *n* = 3 independent biological replicates. (**B** and **D**) Two-way ANOVA, Šidák’s multiple comparisons, *n* = 3 independent biological replicates; data are shown as mean ± SEM.

Previous studies have shown that CoA added to culture media is hydrolyzed extracellularly to 4′-phosphopantetheine, which crosses the membrane and increases intracellular CoA levels (3–5, 28), thereby conferring protection against ferroptosis (3, 5). We recently established that intracellular CoA directly regulates the mitochondrial thioredoxin system by covalently binding to Cys-483 of TXNRD2, thereby enhancing its enzymatic activity (Figure 3A) (5). Upon the depletion of CoA, the impaired TXNRD2 activity compromises the mitochondrial thioredoxin system, leading to the oxidation of its downstream effector peroxiredoxin 3 (PRDX3), as evidenced by a shift from its reduced monomeric form to the oxidized dimer forms (5) (Figure 3A).

Building on these findings, we next investigated whether the CoA-regulated mitochondrial thioredoxin system contributes to the ferroptosis protection by *MESH1* knockdown. To test this, we treated *MESH1*-knockdown HT-1080, RCC4, MDA-MB-231, and A549 cells with ferroptocide (thioredoxin inhibitor) (30) (Figure 3B and Supplemental Figure 3, A and B). Although *MESH1* knockdown protected cells against ferroptosis, this protective effect was abolished by ferroptocide treatment, as quantified by cell viability (Figure 3B and Supplemental Figure 3, A–C), cytotoxicity (Supplemental Figure 3, D and E), or lipid peroxidation measurements (Supplemental Figure 3, F and G). Consistently, treatment with auranofin, an inhibitor of thioredoxin reductase (30), similarly eliminated the protective effect of *MESH1* knockdown (Supplemental Figure 3H). Because both ferroptocide and auranofin target the cytosolic and mitochondrial thioredoxin systems (30) but may exert off-target activities, we next applied a genetic strategy to confirm their target specificity. Specifically, we combined *MESH1* knockdown with siRNA-mediated depletion of either cytosolic thioredoxin (*TXN1*) or mitochondrial thioredoxin (*TXN2*) (Supplemental Figure 3, I and J). Notably, the knockdown of *TXN2*, but not *TXN1*, restored ferroptosis sensitivity in *MESH1*-knockdown HT-1080 cells by cell viability assay (Figure 3C) or lipid peroxidation measurements in MDA-MB-231 cells (Supplemental Figure 3K), demonstrating that the mitochondrial thioredoxin system is required for the ferroptosis protective effect of *MESH1* knockdown.

Because MESH1 is a cytosolic protein (12), our findings imply that CoA must be transported into mitochondria to mediate ferroptosis protection. Since *SLC25A42* encodes the primary mitochondrial CoA transporter (31–33), we examined its role by knocking down *SLC25A42* using 2 independent shRNAs (Supplemental Figure 3L). Notably, the protective effect observed upon *MESH1* knockdown with erastin treatment was abolished by concurrent *SLC25A42* knockdown (Figure 3D). This result confirms the critical role of mitochondrial CoA transport in ferroptosis protection mediated by *MESH1* knockdown (Figure 3D), highlighting the essential role of mitochondrial CoA transport in this process.

We previously demonstrated that imported CoA maintains an active mitochondrial thioredoxin system (5). To investigate whether *MESH1* knockdown influences mitochondrial redox status, we monitored the oxidation state of PRDX3 using its monomer/dimer ratio as a redox indicator (34). Consistent with our previous findings (5), erastin treatment decreased the PRDX3 monomer/dimer ratio in both HT-1080 and RCC4 cells (Figure 3, E and F, and Supplemental Figure 3, M–O), consistent with impaired mitochondrial thioredoxin system due to CoA depletion (5). In contrast, *MESH1* knockdown preserved the PRDX3 monomer/dimer ratio upon erastin treatment, consistent with its role in maintaining intracellular CoA levels and mitigating the mitochondrial thioredoxin system upon CoA depletion by erastin (Figure 3, E and F, and Supplemental Figure 3, M–O).

To further confirm that the protective effects of *MESH1* knockdown on ferroptosis and the PRDX3 oxidative state depend on CoA biosynthesis, we knocked down *COASY*, a key enzyme in CoA biosynthesis, along with *MESH1* in HT-1080 cells (Supplemental Figure 3M). The increased monomer/dimer ratio induced by *MESH1* knockdown was significantly reduced when *COASY* was concurrently knocked down in both HT-1080 and RCC4 cells, indicating that MESH1-regulated PRDX3 activities required CoA biosynthesis (Figure 3, E and F, and Supplemental Figure 3, M–O). Furthermore, we previously found that CoA regulated mitochondrial lipid peroxidation during ferroptosis (5). Using a fluorescent probe specific for mitochondrial lipid peroxidation (35), we observed that erastin dramatically increased mitochondrial lipid peroxidation, which was abolished upon *MESH1* knockdown, mirroring the protective effects of CoA supplementation during erastin treatment (Figure 3G). Taken together, these results indicate that *MESH1* knockdown protects against ferroptosis by preserving intracellular CoA, thereby maintaining the mitochondrial thioredoxin system function and preventing mitochondrial lipid peroxidation.

### MESH1 triggers muscle atrophy by depleting CoA levels in Drosophila.

**Figure 4:**
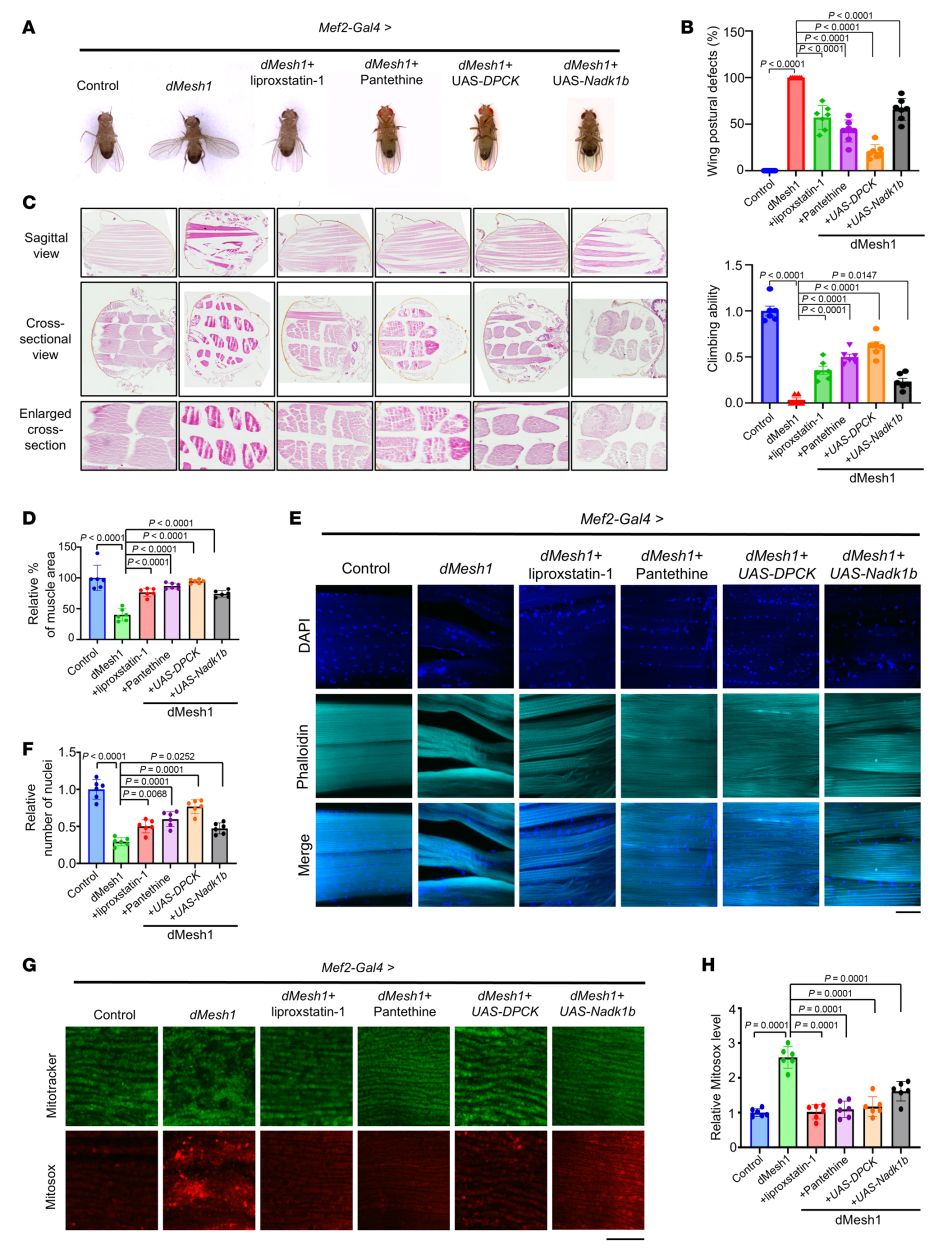
Muscle defects induced by MESH1 overexpression in *Drosophila* were rescued by liproxstatin-1 treatment or by enhancing CoA or NADPH synthesis. Overexpression of *Mesh1* in skeletal muscles resulted in an open wing phenotype (**A**), climbing defects (**B**), muscle shrinkage (**C** and **D**), reduced and clustered nuclei with altered nuclear localization (**E** and **F**), and increased mitochondrial ROS levels (**G** and **H**). These defects were rescued by liproxstatin-1 or pantethine treatment or by overexpression of *DPCK* or *Nadk1b*. Scale bars: 20 μm (**E** and **G**); original magnification, ×4 (**C**). (**B**, **D**, **F**, and **H**) One-way ANOVA, Tukey’s multiple comparisons, *n* = 6 independent biological replicates.

Ferroptosis has been implicated in several skeletal muscle diseases, including cancer cachexia, cancer-induced muscle wasting, and rhabdomyolysis (36–38). A recent study also showed that *Mesh1* is upregulated in response to mitochondrial DNA damage, contributing to ferroptosis and cardiomyopathy (39). Similarly, *MESH1* expression increases during ferroptosis induced by erastin or cystine deprivation (12). Consistent with the protective effect observed in *MESH1*-knockdown cells, we next examined whether elevated MESH1 expression would have the opposite effect. Similarly, in HT-1080 cells, MESH1 overexpression sensitized cells to erastin-induced ferroptosis, an effect reversed by the ferroptosis inhibitor liproxstatin-1 (Supplemental Figure 4A). This finding complements the loss-of-function data, demonstrating that MESH1 levels bidirectionally regulate ferroptosis sensitivity by modulating intracellular CoA metabolism. Given the evolutionary conservation of MESH1 across different species (15), we investigated its physiological role in vivo by selectively modulating *Drosophila* Mesh1 (*dMesh1*) in skeletal muscle. Muscle-specific *dMesh1* knockdown produced no overt phenotype under basal conditions (Supplemental Figure 4B). In contrast, muscle-specific overexpression of *dMesh1* led to abnormal wing posture and impaired climbing ability, indicative of compromised muscle function and reduced strength (Figure 4, A and B). Consistent with enhanced CoA degradation, total CoA levels were significantly reduced in adult fly muscle expressing *dMesh1* (Supplemental Figure 4C).

To exclude the possibility that constitutive *dMesh1* overexpression disrupted muscle development, we employed the *Gal80^ts^* system to temporally control transgene expression (40, 41). By suppressing GAL4 activity during development and inducing *dMesh1* expression exclusively in adult flies, we observed comparable wing positioning and climbing defects. These findings demonstrate that the observed phenotypes are not attributable to developmental abnormalities but instead reflect a postdevelopmental, degenerative effect of *dMesh1* overexpression in mature muscle (Supplemental Figure 4D).

Morphological analyses revealed hallmark features of muscle atrophy, including muscle fiber shrinkage (Figure 4, C and D), decreased nuclear numbers, nuclear clustering, and altered nuclear localization (42) (Figure 4, E and F). Moreover, mitochondrial ROS levels were markedly elevated in *dMesh1*-expressing muscles (Figure 4, G and H), consistent with the established role of CoA in restraining mitochondrial lipid peroxidation. In agreement with this oxidative phenotype, TMRM staining demonstrated a significant reduction in mitochondrial membrane potential, indicating mitochondrial dysfunction in *dMesh1*-overexpressing muscle (Supplemental Figure 4E).

Importantly, lifelong treatment with liproxstatin-1, a ferroptosis inhibitor, markedly rescued the muscle atrophy–associated phenotypes induced by *dMesh1* overexpression (Figure 4, A–E, and Supplemental Figure 4E). In contrast, initiating liproxstatin-1 treatment after the onset of *dMesh1*-driven wing posture and climbing defects failed to reverse these abnormalities, suggesting that ferroptotic damage becomes irreversible once established (Supplemental Figure 4F). This indicates that *dMesh1* promotes ferroptosis in skeletal muscles, thereby contributing to muscle atrophy. Collectively, these data support that elevated MESH1 expression enhances ferroptosis in both experimental models.

CoA biosynthesis is an evolutionarily conserved and fundamental biological pathway (43). In *Drosophila*, hypomorphic mutations in genes essential for CoA biosynthesis (*dPANK/fbl^1^*, *dPPCS^1^,* and *dPPAT-DPCK^43^*) lead to abnormal wing positions, locomotor dysfunction, disrupted lipid homeostasis, increased cell death, and reduced lifespan (43, 44). Consistent with these findings, we observed that muscle-specific knockdown of dephospho-CoA kinase (DPCK), a terminal enzyme in CoA biosynthesis, phenocopied the muscle atrophy caused by *dMesh1* expression (Supplemental Figure 4, G–J). Collectively, these observations highlight the critical role of CoA in muscle maintenance and suggest that CoA depletion is a key contributor to *dMesh1*-mediated muscle pathology.

To test whether *dMesh1* expression triggers muscle defects through CoA depletion, we employed both pharmacological and genetic approaches. First, we supplemented *dMesh1*-expressing flies with pantethine, a CoA biosynthesis precursor (45), and found that it rescued the muscle defects caused by *dMesh1* expression (Figure 4, A–H). Similarly, pantethine supplementation protected HT-1080 cells overexpressing MESH1 from erastin-induced ferroptosis (Supplemental Figure 4A). Next, we coexpressed *DPCK* in muscle to genetically enhance CoA synthesis, which also strongly mitigated *dMesh1*-induced muscle defects (Figure 4, A–H). Together, these results indicate that increasing CoA levels can reverse *dMesh1*-induced muscle atrophy.

Given that MESH1 is a NADPH phosphatase (12) and the knockdown of *Mesh1* protected against ferroptosis by accumulating NADPH, which can be abolished by simultaneous knockdown of *NADK*, a kinase for the synthesis of NADPH from NADH (12), we tested the effects of NADPH on these phenotypes. We found that knockdown of muscular *Nadk1a* expression had no observable effects, whereas *Nadk1b* RNAi expression in muscles induced moderate muscle defects (Supplemental Figure 4, G–J). Importantly, coexpression of *Nadk1b* partially rescued these effects (Figure 4, A–H). These results suggest that *dMesh1*-induced muscle defects are predominantly mediated through CoA depletion and partially through NADPH reduction. In summary, these findings demonstrate that MESH1 overexpression induces ferroptosis in muscle by depleting CoA and, to a lesser extent, NADPH. Restoration of CoA levels — either genetically or pharmacologically — can reverse MESH1-induced muscle atrophy, highlighting CoA as a key metabolic regulator of ferroptosis and muscle integrity.

### MESH1 upregulated in cancer cachexia regulates ferroptosis in mammalian muscle cells.

**Figure 5:**
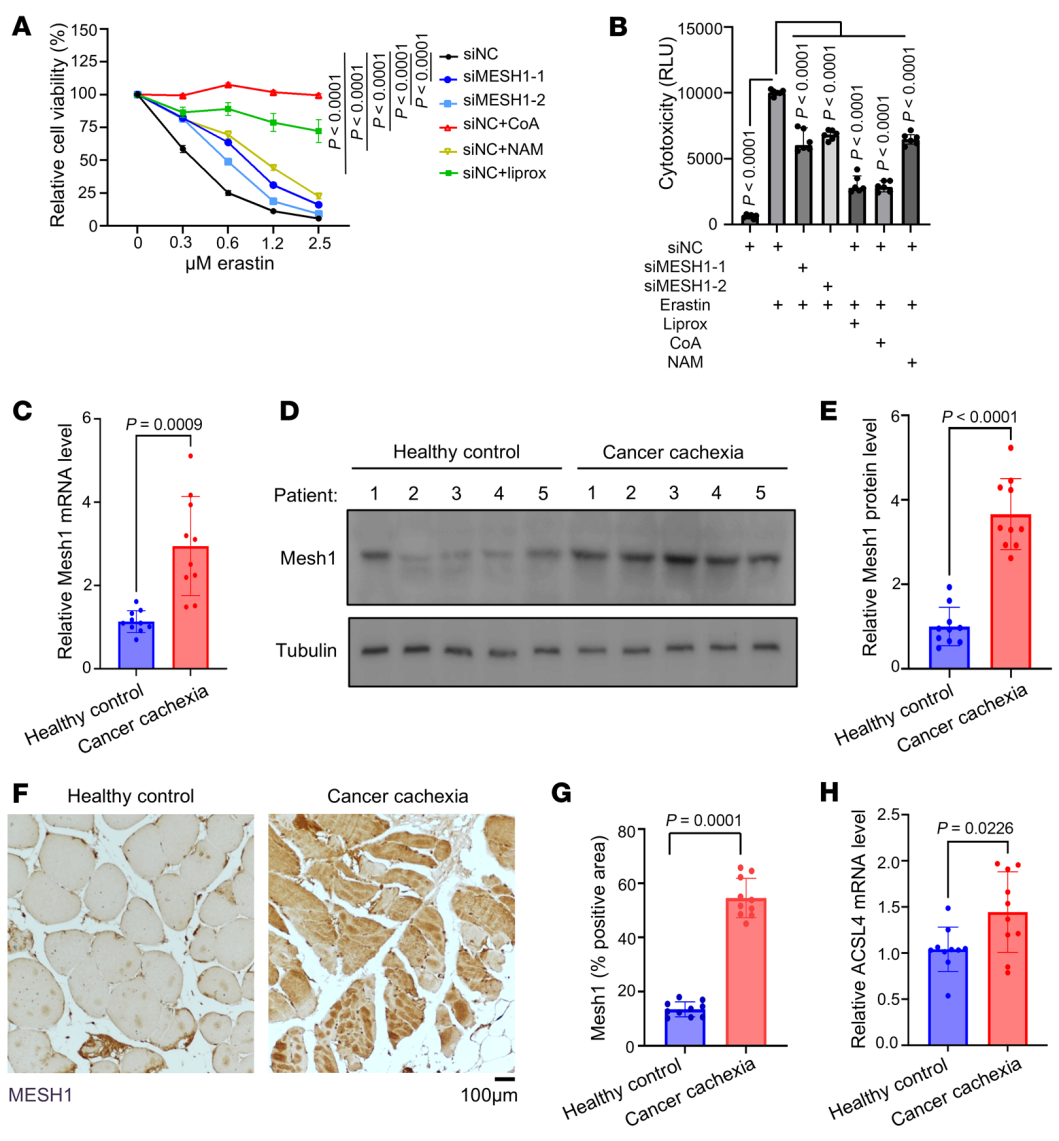
MESH1 is upregulated in cancer cachexia and regulates ferroptosis in mammalian muscle cells. (**A** and **B**) *MESH1* knockdown significantly protected differentiated C2C12 myotubes from erastin-induced ferroptosis, as measured by cell viability (**A**) and cytotoxicity assays (**B**). Supplementation with CoA or NAM, a NAD^+^ precursor that enhances intracellular NADPH levels, similarly protected C2C12 myotubes from ferroptosis, indicating conservation of the MESH1/CoA/NADPH axis in mammalian muscle cells. (**C**) MESH1 mRNA levels were increased in skeletal muscle samples from patients with cancer cachexia compared with healthy controls. (**D** and **E**) Immunoblot analysis (**D**) and quantification (**E**) showing increased MESH1 protein levels in skeletal muscle from patients with cancer cachexia. (**F** and **G**) Immunohistochemical staining (**F**) and quantification (**G**) demonstrating enrichment of MESH1 protein in skeletal muscle sections from patients with cancer cachexia compared with healthy controls. Scale bar: 100 μm. (**H**) Expression of ACSL4, a ferroptosis-associated enzyme, in skeletal muscle from healthy controls and patients with cancer cachexia. (**A**) Two-way ANOVA, Šidák’s multiple comparisons, *n* = 6 independent biological replicates; data are shown as mean ± SEM. (**B**) One-way ANOVA, Tukey’s multiple comparisons, *n* = 6 independent biological replicates. (**C**, **E**, **G**, and **H**) Student’s *t* test, *n* = 10 independent biological replicates; data are shown as mean ± SEM.

To assess whether the MESH1-regulated pathway is conserved in mammalian muscle cells, we examined the role of MESH1 in differentiated C2C12 myotubes under ferroptotic stress. Differentiated C2C12 cells were sensitive to erastin-induced ferroptosis, and *MESH1* knockdown significantly protected these cells from erastin-induced cell death, as measured by both cell viability and cytotoxicity assays (Figure 5, A and B). Consistent with a conserved role for CoA and redox metabolism in ferroptosis, supplementation with either CoA or NAM, a NAD^+^ precursor that enhances intracellular NADPH levels, similarly protected differentiated C2C12 cells from erastin-induced ferroptosis (Figure 5, A and B). These results indicate that the MESH1/CoA/NADPH axis regulating ferroptosis is conserved in mammalian muscle cells.

To assess clinical relevance, we examined MESH1 expression in skeletal muscle samples from healthy controls and patients with rigorously defined cancer cachexia (Supplemental Table 1). Cachexia was confirmed using established clinical and biochemical markers, including elevated C-reactive protein and IL-6 levels and reduced hemoglobin (Supplemental Table 1). Muscle strength and mass were evaluated by handgrip strength (Supplemental Table 1). MESH1 expression was markedly increased in cachectic muscle at both the mRNA and protein levels (Figure 5, C–E). Immunohistochemical analysis further demonstrated significant enrichment of MESH1 protein in muscle sections from patients with cancer cachexia (Figure 5, F and G). Consistent with activation of ferroptotic pathways, we also observed increased expression of ACSL4, a key ferroptosis-associated enzyme, in cachectic muscle (Figure 5H). Together, these findings demonstrate that the MESH1/CoA/NADPH axis is activated in human cancer cachexia and support a conserved role for MESH1-mediated metabolic dysregulation in promoting ferroptotic muscle degeneration.

## Discussion

CoA phosphatase activity was detected in several fractions of a tissue fractionation study more than 50 years ago (9, 46). Since then, several enzymes have been implicated in CoA catabolism. For example, various Nudix hydrolases (NUDT7, NUDT8, and NUDT19) split the CoA molecule into 3′,5′-ADP and phosphopantetheine (46). However, the specific enzyme with CoA phosphatase activity to generate dp-CoA has not been discovered. Our findings indicate that MESH1 functions as a CoA phosphatase that dephosphorylates CoA to generate dp-CoA. MESH1 plays a role in degrading CoA levels, which contributes to the regulation of ferroptosis. Interestingly, our previous studies have shown that MESH1 is an NADPH phosphatase that regulates NADPH levels under the stress of ferroptosis (12). Therefore, NADPH and CoA preservation upon *MESH1* knockdown contribute to the protection of ferroptosis. While reducing the NADPH or CoA only partially mitigated the ferroptosis protection, the combined effects of both metabolites abolished the protection effects completely (Figure 2H). Therefore, such a role of MESH1 in both branches of ferroptosis protection may explain why MESH1 removal had a strong ferroptosis protection capacity. It is interesting to note that the blockage of xCT and cystine import by xCT inhibitors (such as erastin) depleted both GSH and CoA as cysteine contributes to the synthesis of both ferroptosis-protecting metabolites. By affecting NADPH and CoA, MESH1 regulates both arms of the xCT-dependent ferroptosis protectants, either directly (CoA) or indirectly (NADPH-driven GSH regeneration).

While CoA supplementation was found to replenish depleted intracellular CoA levels and robustly protect against ferroptosis (3–5), the underlying mechanism remains poorly defined. Previously, we identified the thioredoxin systems essential for CoA-mediated ferroptosis protection, and CoA regulates the activity of the mitochondrial thioredoxin system via CoAlation of TXNRD2 (5). Consistent with the regulation of CoA, we found that ferroptosis protection upon *MESH1* knockdown requires a functional mitochondrial thioredoxin system. CoA levels were reported to be much higher in mitochondria than in cytosol (47). Given that MESH1 is a cytosolic protein (12), ferroptosis protection mediated by *MESH1* knockdown preservation of cytosolic CoA levels by preventing its degradation, thereby enabling its transport into mitochondria to maintain a functional mitochondrial thioredoxin system. Consistent with this idea, the ablation of the mitochondrial CoA transporter (*SLC25A42*) abolished the ferroptosis protection of *MESH1* knockdown (Figure 3D). Therefore, our findings suggest the CoA transport between different cellular compartments in fine-tuning the response to ferroptosis. These mechanistic insights have direct relevance to cancer cachexia, a clinical syndrome characterized by progressive skeletal muscle loss accompanied by systemic inflammation. The upregulation of MESH1 in cachectic human muscle, together with increased expression of the ferroptosis-associated enzyme ACSL4, is consistent with activation of a pro-ferroptotic state in vivo. We propose that MESH1 upregulation may predispose cachectic muscle to lipid peroxidation and degenerative remodeling. Collectively, these human observations complement our functional data showing that perturbation of the MESH1/CoA/NADPH axis modulates ferroptosis sensitivity in muscle models, and they implicate MESH1-linked redox failure as a potential contributor to human muscle wasting.

SpoT plays a role in the bacterial stringent response by hydrolyzing alarmone (p)ppGpp, the central metabolite that accumulates during metabolic stresses to coordinate a stringent response. While MESH1 also shared (p)ppGpp hydrolase activities (11), the levels of (p)ppGpp in metazoans were either absent or low (11, 19, 20), which prompted us to identify alternative substrates in human cells. After we found NADPH as the relevant substrate of MESH1, another study found that a bacterial orphan, short alarmone hydrolase (SAH), in the phytopathogen *Xanthomonas campestris* pv. *campestris* (XccSAH) also exhibits NADPH phosphatase activity and is critical for the production of NADH by XccSAH (17). Therefore, there may be significant conservation between the enzymatic activities of bacterial SAH/SpoT and MESH1. Given our discovery of MESH1 as a CoA phosphatase, bacterial SAH and SpoT may also have similar enzymatic activity to regulate CoA levels/metabolism in bacteria. This possibility may suggest a function of SAH/SpoT in regulating metabolisms via a mechanism distinct from (p)ppGpp, a possibility that will be explored in the future.

While this study successfully identified MESH1 as a CoA phosphatase regulating ferroptosis, several limitations should be acknowledged. First, although our in vitro and in vivo models provided clear evidence of MESH1’s role in CoA metabolism, the extent to which these findings translate to other physiological or pathological conditions, particularly in human tissues beyond the cell lines and *Drosophila* models used, remains to be further investigated in the future. Second, we have previously found that MESH1 removal triggered many features of the bacterial stringent response (12, 13, 15), and the relative contribution of CoA and NADPH phosphatase activities to these phenotypes has not been fully characterized. In addition to ferroptosis, MESH1 may play additional roles in other physiological and pathological adaptions. Future studies will address these knowledge gaps and assess MESH1’s role across a broad range of cell types and stress conditions in mammalian systems and different disease settings.

## Methods

### Sex as a biological variable.

Human muscle samples were obtained from both male and female subjects. Cell line and *Drosophila* experiments were not designed to evaluate sex as a biological variable. No sex-dependent differences were specifically observed, and analyses were not powered to detect such differences.

### Purification of hMESH1.

hMESH1 was codon optimized for *E*. *coli* expression, and the gene was synthesized and cloned into a modified pET28a vector using in-fusion cloning with the Hi-Fi DNA Assembly kit (New England Biolabs) to create hMESH1 with a N-terminus fusion of a His^10^-SUMO tag. The vector was transformed into BL21 cells that were grown at 37°C until the cells reached an OD_600_ of 0.5, at which point, 1 mM isopropyl β-d-1-thiogalactopyranoside (GoldBio) was added for 2 hours of expression. The cells were lysed using a French press at 1,200 psi. The target protein was purified using Ni affinity chromatography (Cytiva), and the His^10^-SUMO tag was cleaved using SENP1 protease. hMESH1 was further purified via an additional round of Ni affinity chromatography and size-exclusion chromatography using the Superdex 75 column (GE Life Sciences) in a buffer containing 200 mM NaCl, 50 mM Tris, pH 8, and 0.1% β-mercaptoethanol.

### Enzymology.

Enzyme assays were performed with a buffer containing 200 mM NaCl, 50 mM Tris, pH 8, and 1 mM MnCl_2_. For measuring the enzyme kinetics of CoA and acetyl-CoA, a final concentration of 200 nM hMESH1 was mixed with serial dilutions of CoA (Thermo Fisher Scientific) and acetyl-CoA (Sigma-Aldrich) starting at a final concentration of 1.5 mM, and the reaction was run at 37°C. Data were collected at 1, 2, 3, 4, 5, and 6 min time points, and the reaction was quenched by the addition of 5 M formic acid (Sigma-Aldrich). The level of free phosphate released during the enzymatic reaction was calculated using malachite green assay (Sigma-Aldrich) following the manufacturer’s instructions and measuring absorbance at 620 nm. The *K_m_* and *V_max_* values were calculated using the Michaelis-Menten equation. To calculate the specific activity of the various CoA species, a final concentration of 200 nM hMESH1 was mixed with a final concentration of 0.5 mM CoA (Thermo Fisher Scientific), acetyl-CoA (Sigma-Aldrich), malonyl-CoA (Sigma-Aldrich), succinyl-CoA (Sigma-Aldrich), HMG-CoA (Sigma-Aldrich), octanoyl-CoA (Sigma-Aldrich), and palmitoyl-CoA, and the enzymatic reaction ran for 6 min.

### Mass spectrometry for CoA/acetyl-CoA dephosphorylation assay.

For validating the product of the enzymatic reaction, 1.5 mM CoA or acetyl-CoA was incubated with 33 μM hMESH1 or buffer for 2 hours at 37°C in a buffer consisting of 200 mM NaCl, 50 mM Tris, pH 8, and 1 mM MnCl_2_. CoA/acetyl-CoA, dp-CoA, and dp-acetyl-CoA were quantified using our previously described LC-MS/MS method (48) with modifications. Prepared samples were dissolved in 100 μL of mobile phase A (2% acetonitrile in 100 mM ammonium formate, pH 5.0), and a 40 μL aliquot was injected onto an Agilent ZORBAX 300SB-C8 column (100 × 2.1 mm, 3.5 μm), equipped with a ZORBAX guard column (12.5 × 2.1 mm, 5 μm), using an ExionLC AD system (Sciex). The column oven and autosampler temperatures were set to 42°C and 5°C, respectively. The LC method operated at 0.2 mL/min with the following gradient: 100% mobile phase A (0% mobile phase B: 98% acetonitrile in 5 mM ammonium formate, pH 6.3) for 2 min. Mobile phase B increased to 60% over 8 min. Mobile phase B further increased to 90% in 1 min and held for 19 min. The gradient returned to the initial condition in 1 min, followed by 10 min of reequilibration before the next injection. 

The liquid chromatograph was coupled to a 6500^+^-QTRAP mass spectrometer (Sciex) in positive ionization mode, with the following source settings: turbo ion spray source, 500°C; N_2_ nebulization, 65 psi; N_2_ heater gas, 55 psi; curtain gas, 30 psi; collision-activated dissociation gas pressure, high; turbo ion-spray voltage, 5,500 V; declustering potential, 90 V; entrance potential, 10 V; collision energy, 50 V; collision cell exit potential, 10 V; and precursor ion (Q1) range, *m*/*z* 768–1,100, with product ions (Q3) calculated as Q1 − 507. For acyl-dp-CoAs, a separate MRM method was used with Q1 ions ranging from *m*/*z* 688 to 988 and Q3 ions calculated as Q1 − 427. Data acquisition was performed using Analyst software (version 1.6.1; Sciex). 

### Chemicals.

The following chemicals were used: ferroptocide (F1293, TCI), PANKi (31002, Cayman), CoA (F15115, Astatech), erastin (5499, Bio-techne), ferrostatin-1 (17729, Cayman), liproxstatin-1 (17730, Cayman), deferoxamine (D9533, Sigma-Aldrich), NAC (A9165, Sigma-Aldrich), and PANKi (Cayman, 31002).

### Cell culture.

HT-1080, HEK293T, MDA-MB-231, A549, and RCC4 cell lines were obtained from the Cell Culture Facility at Duke University. Prior to cryopreservation, the identity of the cell lines was confirmed by short tandem repeat profiling, and they were verified as free of mycoplasma contamination by the facility. The cells were cultured for less than 6 months. They were maintained in a humidified incubator at 37°C with 5% CO_2_ in DMEM (Gibco, 11995), supplemented with 10% heat-inactivated FBS (10082147, Thermo Fisher Scientific) and antibiotics (10,000 U/mL streptomycin and 10,000 U/mL penicillin; 15140122, Thermo Fisher Scientific).

### Constructs and lentivirus viral infections.

siRNAs designed to target human *MESH1*, *COASY*, *TXN1*, *TXN2*, and *SLC25A42* were sourced from Dharmacon (catalog D-031786-01, D-006751-01, M-006340-01, M-017448, and D-007361-03) and Qiagen (SI04167002 for MESH1-02 targeting). Unless otherwise stated, siMESH1 refers to MESH1-01. The cDNA for MESH1-WT (NCBI RefSeq NM_001286451.1) was inserted into the pLX302 lentiviral vector using Gateway cloning techniques. To generate the MESH1-E65A mutant, a site-directed mutation was introduced into the MESH1-WT clone using the QuikChange II XL site-directed mutagenesis kit (200521, Agilent). Lentiviral particles were produced by transfecting HEK293T cells in 6-well plates using a 1:1:0.1 ratio of lentiviral vector, pMD2.G, and psPAX2, facilitated by the TransIT-LT1 transfection reagent (Mirus). The resulting lentivirus was passed through a 0.45 μm cellulose acetate filter (28145-481, VWR), and 250 μL of the virus-containing media was added to a 60 mm dish of target cells, along with polybrene (8 μg/mL), followed by puromycin selection.

### Cell viability and cytotoxicity.

Cell viability was evaluated using the CellTiter-Glo luminescent assay (Promega), following the protocol provided by the manufacturer. In brief, 15 μL of the CellTiter-Glo reagent was added to cells in a 96-well plate containing 100 μL of media, and the mixture was shaken for 10 min. Luminescence was then measured with a plate reader. For assessing cell death, the CellTox Green assay (Promega) was used. The dye was diluted 1:1,000 in the media, and fluorescence was measured to quantify cell death using a fluorescence plate reader.

### Western blots.

Protein concentration was determined using the BCA assay (23227, Thermo Fisher Scientific). After extraction, the proteins were resolved on 12% SDS-PAGE gels under nonreducing or reducing conditions, transferred onto a PVDF membrane, and blocked with 5% nonfat milk in 1× TBST. Membranes were incubated overnight at 4°C with the following primary antibodies: MESH1 (1:1,000, HPA040895, Sigma-Aldrich; 1:2,500, 21091-1-AP, ProteinTech), PRDX3 (1:1,000, 10664-1-AP, Thermo Fisher Scientific), GAPDH (1:2,000, sc-25778, Santa Cruz Biotechnology), TXNRD2 (1:1,000, PA529458, Thermo Fisher Scientific), and α-tubulin (1:1,000, sc-32293, Santa Cruz Biotechnology). TXNRD2 was purified from HT-1080 cells overexpressing TXNRD2-v5 using the V5-tagged Protein Purification Kit Version 2 (3317, MBL). For TXNRD2, cells were lysed in NP-40 buffer containing 25 mM *N*-ethylmaleimide (NEM, 23030, Thermo Fisher Scientific) and protease inhibitor (04693116001, Roche) at 4°C with constant agitation, followed by centrifugation at 21,000*g* for 10 min. The supernatant was purified using the V5-tagged Protein Purification Kit. For PRDX3 blots, cells were washed with PBS and incubated in NEM buffer (40 mM HEPES, pH 7.4, 50 mM NaCl, 1 mM EDTA, 1 mM EGTA, 100 mM NEM, and protease inhibitor) for 10 min. Cells were then lysed with 1% CHAPS for protein quantification.

### RT-qPCR.

RNA was extracted and purified using the RNeasy Mini Kit (Qiagen) according to the manufacturer’s guidelines. Reverse transcription was carried out with random hexamers and SuperScript IV reverse transcriptase (Invitrogen). For RT-qPCR, the resulting cDNA was mixed with primers and Power SYBR Green PCR Mix (Applied Biosystems) and analyzed using the StepOnePlus Real-time PCR system (Applied Biosystems). All samples were run in triplicate to calculate the mean ± SEM. Data shown are representative of at least 2 independent experiments. Primer sequences are available in Supplemental Table 2.

### Lipid peroxidation assay.

Lipid peroxidation was evaluated using C11-BODIPY staining, following the manufacturer’s protocol (D3861, Thermo Fisher Scientific). Cells were treated with either vehicle or specific compounds for 16 hours. Afterwards, cells were incubated with a 10 μM C11-BODIPY solution for 1 hour and then harvested, washed, and resuspended in PBS containing 1% BSA. Lipid peroxidation was measured using flow cytometry (FACSCanto TM II, BD Biosciences). For assessing mitochondrial lipid peroxidation, cells were incubated for 30 min with mitoPerOx (200 nM, 18798, Cayman) (35).

### Drosophila husbandry and stocks.

Flies were maintained at 25°C with 60% humidity on a 12-hour light/dark cycle and reared on standard cornmeal, soy flour, yeast, and agar medium. Fly stocks were obtained from the Bloomington Drosophila Stock Center (BDSC), Vienna Drosophila Resource Center (VDRC), and FlyORF. The following stocks were used: *Mef2-Gal4* (BDSC 27390), *Gal80^ts^* (BDSC 7019), *UAS-dMesh1* (BDSC 22234), *UAS-Nadk1b* (BDSC 59052), *Dpck-RNAi* (BDSC 57529), and *Nadk1a-RNAi* (BDSC 57753) from BDSC; *Dpck-RNAi* (v100276), *Nadk1a-RNAi* (v104271), and *Nadk1b-RNAi* (v41825) from VDRC; and *UAS-Dpck* (F003172) from FlyORF.

### Drosophila climbing assay analysis.

Flies expressing *dMesh1* in muscles (*Mef2-Gal4 > dMesh1*) were raised on fly food supplemented with either liproxstatin-1 (1 mg/mL) or pantethine (1.6 mg/mL) and subjected to a climbing assay. For the assay, 20 F1 flies were transferred to an empty vial marked at 5 cm. After gently tapping them to the bottom, flies were given 8 s to climb. The percentage of flies crossing the 5 cm mark was recorded. Each assay was performed in technical triplicates with a 1 min rest between replicates.

### H&E staining.

For H&E staining, flies were dissected and fixed in 10% formalin (VWR, catalog 11699404) overnight, followed by dehydration, paraffin embedding, and sectioning into 5 μm slices. Sections were deparaffinized and stained with hematoxylin (catalog GHS232, Sigma-Aldrich) and eosin (catalog HT110116, Sigma-Aldrich) following a standard protocol. Imaging was performed using a Nikon Eclipse Ni-E upright fluorescence microscope with a ×4 objective.

### CoA quantification.

Total CoA levels in *Drosophila* muscle were measured using the Coenzyme A Assay Kit (MAK504, Sigma-Aldrich) according to the manufacturer’s instructions. For each biological replicate, 20–30 adult thoraces were dissected in ice-cold PBS and immediately snap-frozen. Tissues were homogenized in CoA Assay Buffer on ice and deproteinized. Clarified supernatants were subjected to fluorometric detection according to the kit protocol. Fluorescence was measured at excitation/emission of 535/587 nm using a microplate reader. CoA concentrations were calculated from a standard curve and normalized to total protein content determined from parallel samples.

### Immunofluorescence staining and confocal microscopy.

For immunostaining of *Drosophila* indirect flight muscles, thoraxes were dissected and fixed in 4% paraformaldehyde (catalog 15710, Electron Microscopy Sciences) overnight at 4°C. The thoraces were bisected along the sagittal axis and incubated with Alexa Fluor 633 Phalloidin (catalog A22284, Invitrogen) to visualize F-actin. Nuclei were counterstained with DAPI for 10 min. The samples were mounted onto glass slides using an antifade mounting medium and imaged using a Zeiss LSM710 confocal microscope. Images were acquired and processed using ZEN software.

### MitoSOX and TMRM staining assay.

Flies were anesthetized, and hemithoraces were dissected in *Drosophila* Schneider’s Medium (DSM) (Thermo Fisher Scientific). Hemithoraces were then incubated in DSM containing 5 μM MitoSOX Red (Thermo Fisher Scientific, M36008) with 100  nM MitoTracker Green (Thermo Fisher Scientific, M7514) or 1 μM TMRM (Abcam, ab228569-1001). Staining was done for 20  min at room temperature, and then the samples were rinsed twice for 30 s each wash with DSM. Samples were immediately mounted in DSM and imaged within 20 min using identical confocal microscope settings across all groups.

### Statistics.

All experiments were performed using independent biological replicates, as indicated in the figure legends. Biological replicates refer to experiments conducted on separate days using independently prepared cells. Data are presented as mean ± SEM or mean ± SD as specified.

For datasets with *n* ≥ 5, data distribution was assessed using the Shapiro-Wilk normality test in GraphPad Prism. When datasets did not significantly deviate from normal distribution (*P* > 0.05), parametric statistical tests were applied. For comparisons between 2 groups, a 2-tailed unpaired Student’s *t* test was used. For multiple group comparisons, 1- or 2-way ANOVA followed by Šidák multiple-comparison test was performed.

For experiments with *n* = 3 biological replicates, formal assessment of normality was underpowered. These experiments represent independent biological repeats rather than technical replicates and were reproduced across multiple experimental systems. When variance was comparable between groups and data distribution appeared approximately symmetric without evident outliers, parametric tests were applied. In representative datasets, nonparametric analyses (Mann-Whitney test for 2-group comparisons or Kruskal-Wallis test for multiple comparisons) were additionally performed to evaluate robustness. When nonparametric testing did not reach statistical significance with *n* = 3 but trends and data distribution remained consistent, parametric analyses were reported.

Key findings were validated across multiple independent cell lines and orthogonal assays, including cell viability, cytotoxicity, and lipid peroxidation measurements, to ensure reproducibility. Statistical tests used for each experiment are specified in the corresponding figure legends. Statistical significance was defined as *P* < 0.05. All analyses were performed using GraphPad Prism software.

### Study approval.

This study included patients undergoing orthopedic surgery at Singapore General Hospital between July 2023 and November 2025. Gluteus maximus skeletal muscle samples were collected intraoperatively. Cancer cachexia was defined according to established international consensus criteria. Written informed consent was obtained from all participants prior to sample collection. The study was approved by the SingHealth Centralised Institutional Review Board (reference 2023/2271).

### Data availability.

All data and reagents supporting the results of this study will be made available by the authors upon reasonable request. Values for all data points in graphs are reported in the Supporting Data Values file.

## Author contributions

CCL, HWT, PZ, and JTC conceived the experiments and wrote the manuscript. CCL performed the majority of the experiments. JR and AAM performed biochemical studies. HWT, PZ, and JTC supervised the work. CKCD, SYC, SMC, KYG, WJ, WXL, QJ, Yanting Chen, TS, JW, Yueqi Chen, YO, and PJ collaborated in the discussion and experiments. JH, KC, MCF, and GFZ provided critical feedback.

## Conflict of interest

The authors have declared that no conflict of interest exists.

## Funding support

This work is the result of NIH funding, in whole or in part, and is subject to the NIH Public Access Policy. Through acceptance of this federal funding, the NIH has been given a right to make the work publicly available in PubMed Central.

- Duke DCI Pilot Project (to PZ and JTC).
- Duke SOM Discovery Fund (to PZ and JTC).
- Department of Defense grants W81XWH-17-1-0143, W81XWH-15-1-0486, W81XWH-19-1-0842, and W81XWH-20-1-0907 (to JTC).
- NIH grants R01GM124062 (to PZ and JTC) and R21AI149205 (to JTC).
- Singapore Ministry of Education AcRF Award FY2025-MOET1-0004 (to HWT).
- Diana Koh Innovative Cancer Research Award (Duke-NUS-DKICRA/2024/0001) (to HWT).
- National Medical Research Council grants MOH-001208-00, MOH-001885-00, and MOH-001831-00 (to HWT).

## Supplementary Material

Supplemental data

Unedited blot and gel images

Supporting data values

## Version 1 04/25/2026

In-Press Preview

## Version 2 06/01/2026

Electronic publication
