# Supplementary material for: MESH1-mediated coenzyme A degradation drives ferroptosis sensitivity and muscle pathology
Source: J Clin Invest. 2026 Apr 25;136(11):e202212. doi: 10.1172/JCI202212 (PMC13221220; doi:10.1172/JCI202212)
Supplement: Supplemental data [file jci-136-202212-s060.pdf]

Supplemental information for

MESH1-mediated Coenzyme A degradation drives ferroptosis sensitivity and muscle pathology

Chao-Chieh Lin[1,2*], Joshua Rose[3*], Alexander A Mestre[1,2,3*], Chien-Kuang Cornelia Ding[1,2], Ssu-Yu Chen[1,2], Sze Mun Choy[9], Kah Yong Goh[9], Weiyi Jiang[9], Wen-Xing Lee[9], Qizhou Jiang[9], Yanting Chen[7], Tianai Sun[1,2], Jianli Wu[1,2], Yueqi Chen[8], Yunju Oh[8], Pyeonghwa Jeong[8], Jiyong Hong[6,8], Kenon Chua[10], Michael C. Fitzgerald[8], Guo-Fang Zhang[4,5], Hong-Wen Tang[9†], Pei Zhou[3†], Jen-Tsan Chi[1,2†]

Corresponding author:
Jen-Tsan Ashley Chi; jentsan.chi@duke.edu
Pei Zhou; peizhou@biochem.duke.edu
Hong-Wen Tang; hongwen.tang@duke-nus.edu.sg

A

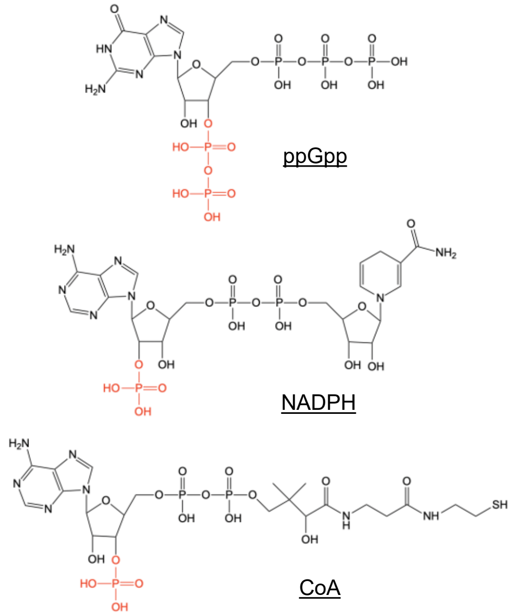

B

	K_{cat} (s^{-1})	K_M (mM)	K_{cat}/K_M (s^{-1} mM^{-1})
ppGpp	36.6 +/- 0.55	3.87 +/- 0.59	9.46
NADPH	1.76 +/- 0.18	0.12 +/- 0.01	14.4 +/- 1.1
CoA	0.54 +/- 0.1	0.17 +/- 0.04	3.3 +/- 0.8

Supplemental Fig. 1 Structural rationale and cross-substrate kinetics for MESH1.
(**A**) Comparison of the chemical structures of (p)ppGpp, NADPH, and CoA. The phosphate group that is hydrolyzed by MESH1 is shown in red. (**B**) A comparison of the kinetic properties of ppGpp, NADPH, and CoA. The ppGpp kinetic data was calculated by Sun et al, and the NADPH calculations were calculated in Ding et al (10, 11).

Supplemental Figure 2

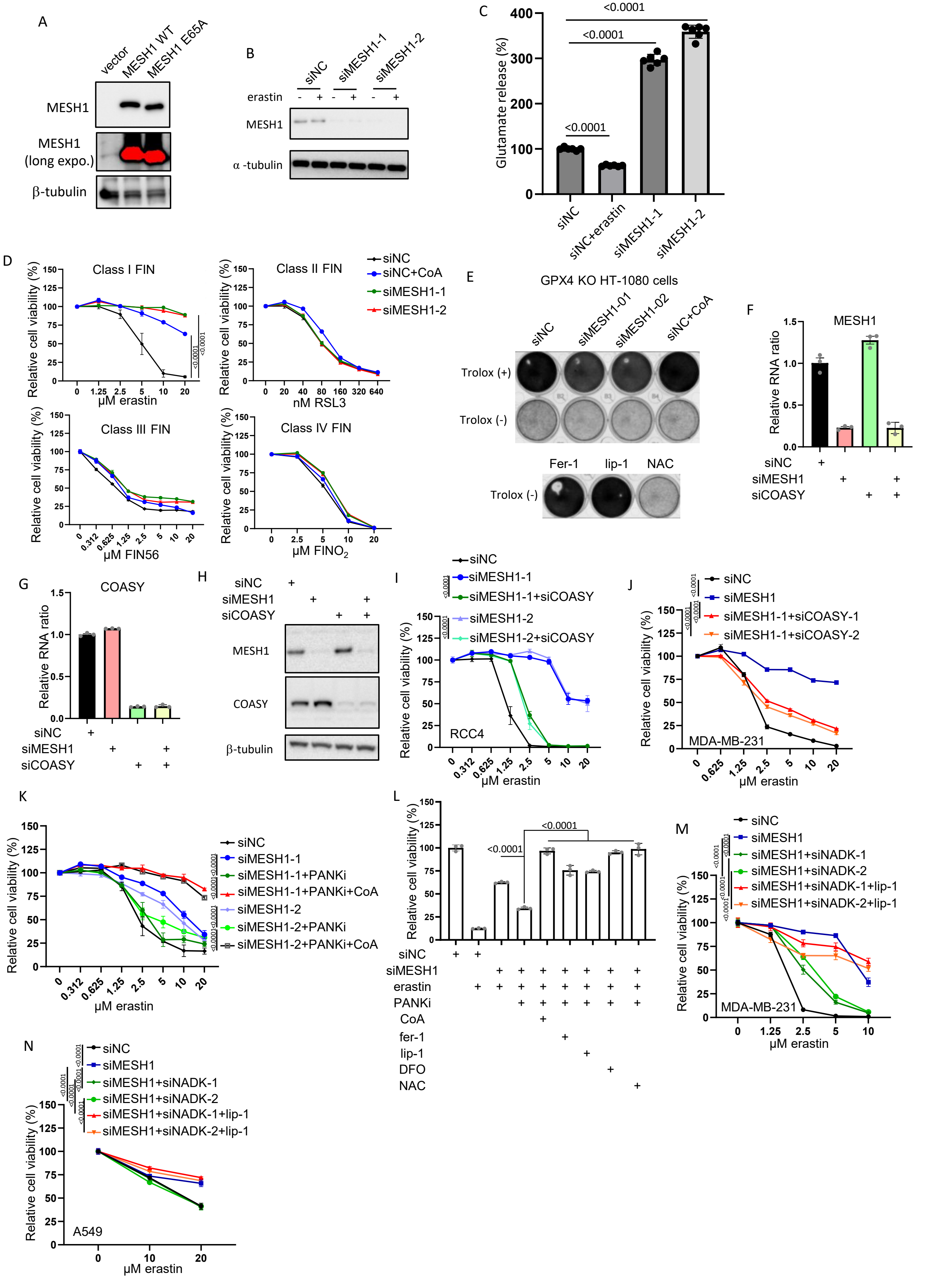

Supplemental Figure 2

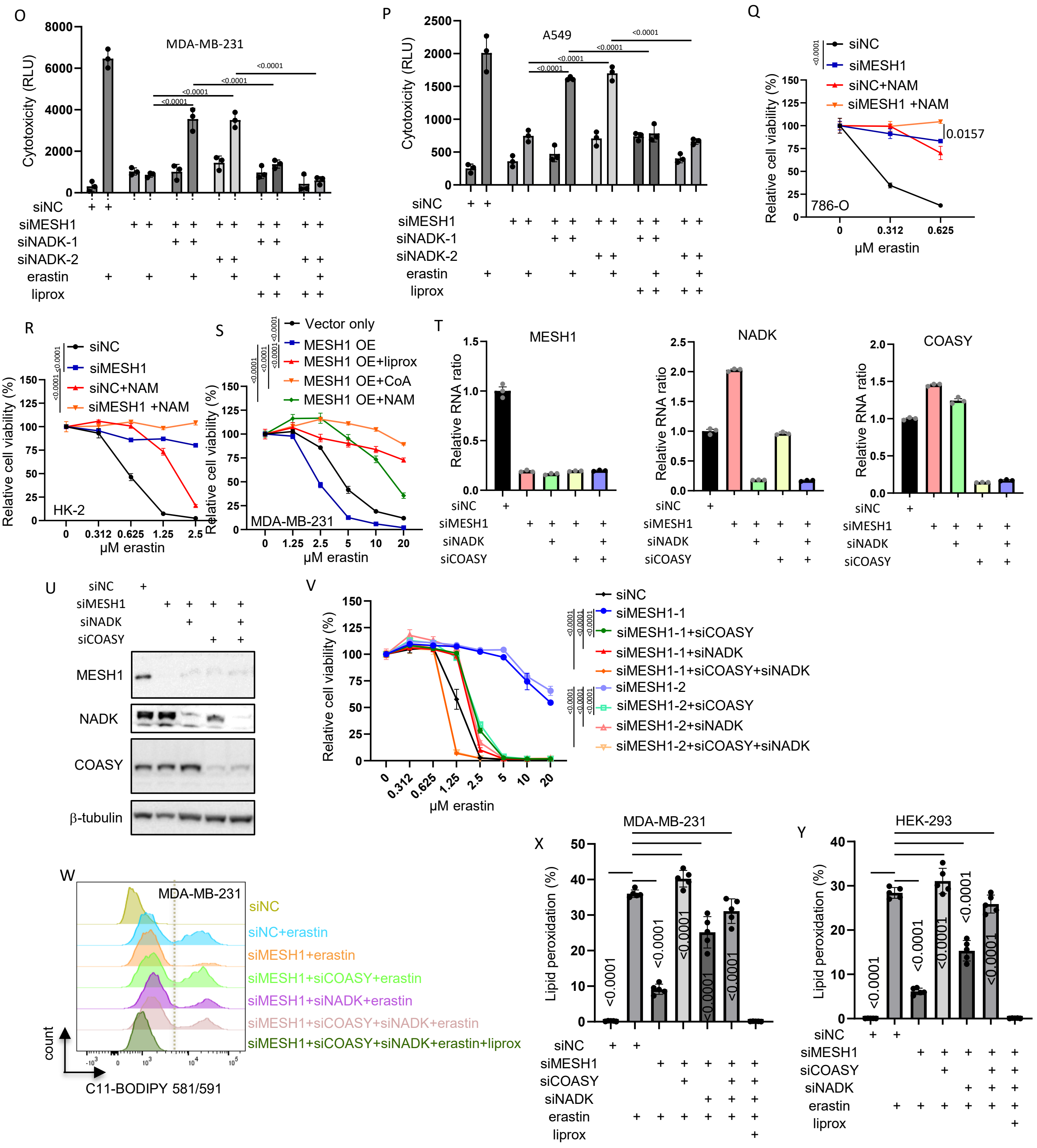

Supplemental Fig. 2 CoA biosynthesis underlies protection by MESH1 knockdown and is specific to class-I FINs.
(**A**) Western blots confirmed MESH1 expression in HEK-293T cells over-expressing empty vector, wild-type MESH1 (MESH1-WT), and the catalytically-null mutant (MESH1-E65A). b-tubulin, loading control. (**B**) Western blots validated the knockdown of MESH1 in HT-1080 cells with the transfection of control or two independent MESH1 siRNAs. α-tubulin, loading control. (**C**) Knockdown of MESH1 using two independent siRNAs in HT-1080 cells increased xCT activity, as quantified by measuring glutamate release into the culture medium. Erastin (20 μM, 2 hours) was used as a positive control for xCT inhibition. (**D**) MESH1 knockdown or COA supplement only rescued ferroptosis, triggered by class I ferroptosis inducer in HT-1080 cells. (**E**) MESH1 knockdown or CoA supplement did not rescue GPX4 knockout induced ferroptosis. GPX4 KO HT-1080 cells maintained in Trolox were transfected with two independent MESH1 siRNA or CoA supplement for 48 hours, before Trolox removal for 24 hours. The cell viability was visualized by crystal violet staining. Ferroptosis inhibitors: ferrostatin-1 (Fer-1, 10 μM); liproxstatin-1 (lip-1, 2 μM); N-acetyl cysteine (NAC, 500 μM). CoA, 100 μM. (**F-H**) Validation of MESH1 and COASY siRNA knockdown by RT-PCR (**F-G**) or Western blots (**H**) in HT-1080 cells. (**I-J**) CoA biosynthesis is required for the protective effect of MESH1 knockdown upon erastin treatment. RCC4 (**I**) and MDA-MB-231 cells (**J**) were transfected with two independent MESH1 siRNA (**I**) or in combination with two independent COASY siRNA (**J**) for 2 days, subjected for 24 hours of erastin treatment, and quantified by Cell-Titer Glo assay. (**K**) PANKi (5μM, chemical inhibitor of pantothenate kinase) abolished the protective effects of two independent MESH1 siRNAs by inhibiting CoA biosynthesis upon erastin treatment in RCC4 cells. (**L**) PANKi (5 μM) abolished the protective effects of MESH1 knockdown in RCC4 cells by ferroptosis. CoA (100 μM); ferrostatin-1 (Fer-1, 10 μM); liproxstatin-1 (lipro, 2 μM); deferoxamine (DFO, 80 μM); N-acetyl cysteine (NAC, 500 μM). (**M–P**) Concurrent knockdown of MESH1 and NADK using two independent siRNAs abolished the protective effect of MESH1 knockdown against ferroptosis in MDA-MB-231 (**M, O**) and A549 cells (**N, P**), as quantified by cell viability (**M, N**) and cytotoxicity assays (**O, P**). Liproxstatin-1 (Lip-1) was used to confirm ferroptotic cell death. (**Q–S**) Nicotinamide (NAM, 20 mM), a cell-permeable NAD^+ precursor, further potentiated the protective effect of MESH1 knockdown in 786-O (**Q**) and HK-2 cells (**R**). NAM supplementation also attenuated the ferroptosis-promoting effect of MESH1 overexpression in MDA-MB-231 cells (**S**). (**T-U**) Validation of MESH1, COASY, and NADK siRNA knockdown in HT-1080 cells by RT-PCR (**T**) or Western blots (**U**). (**V-Y**) Double knockdown of NADK (NADPH synthesis) and COASY (CoA synthesis) fully abolished the protective effects of two independent MESH1siRNAs in RCC4 cells as quantified by cell viability assay (**V**), or lipid peroxidation measurements in MDA-MB-231 cells (**W-X**), or HEK-293 cells (**Y**). (**C,L,X,Y**) One-way ANOVA, Tukey's multiple comparisons. (**O,P,Q,R,S,V**) Two-way ANOVA, Sidak's multiple comparisons. (**D,F,G,I,K,L,M,N,O,P,Q,R,V**) n = 3 independent biological replicates. (**C,J,S**) n = 6 independent biological replicates. (**X,Y**) n = 5 independent biological replicates. Data represent mean ± SEM.

Supplemental Figure 3

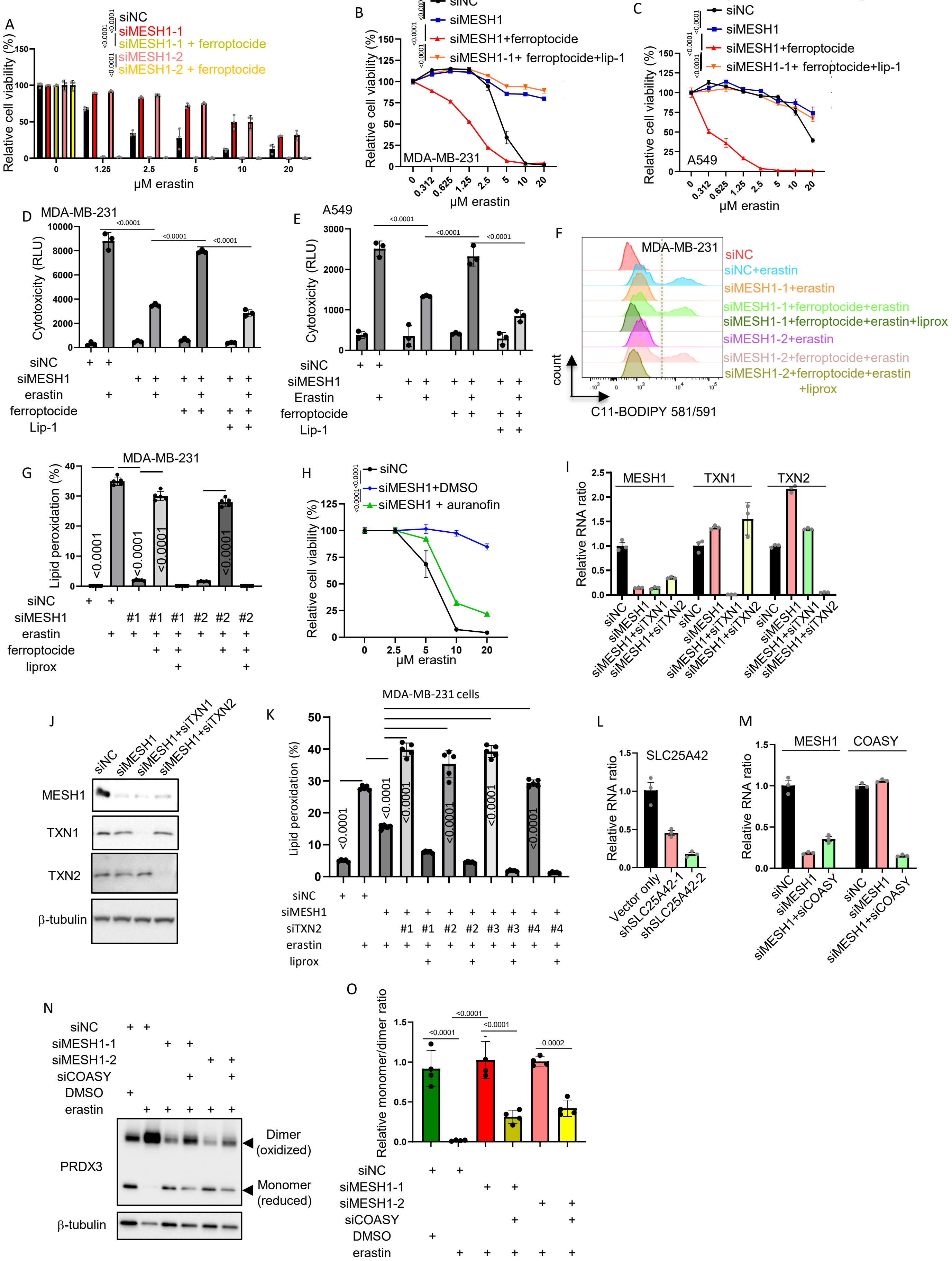

Supplemental Fig. 3 Protection by MESH1 knockdown requires the mitochondrial thioredoxin system and SLC25A42. (**A-F**) Ferroptocide (2μM, chemical inhibitor of thioredoxin) abolished the protective effects of MESH1siRNA upon erastin treatment in RCC4 (**A**), MDA-MB-231 (**B,D,F,G**), or A549 cells (**C,E**). These cell lines were transfected with control or MESH1 siRNA for 2 days and erastin treatment for 18 hours for Cell-Titer Glo assay (**A-C**), cytotoxicity assay (**D,E**) or lipid peroxidation measurement (**F-G**). Liproxstatin-1 (Lip-1) was used to confirm ferroptotic cell death. (**H**) Auranofin (1.5 μM, chemical inhibitor of thioredoxin reductase) abolished the protective effects of MESH1 knockdown upon erastin treatment. HT-1080 cells were transfected with control or MESH1 siRNA for 2 days and erastin treatment for 20 hours for Cell-Titer Glo assay. (**I-J**) Validation of MESH1, TXN1, and TXN2 siRNA knockdown in HT-1080 cells by RT-PCR (**I**) or Western blots (**J**). (**K**) Double knockdown of MESH1 and TXN2 using four independent TXN2 siRNAs abolished the protective effect of MESH1 knockdown, as quantified by lipid peroxidation measurements in MDA-MB-231 cells. (**L**) Validation of SLC25A42 knockdown in HT-1080 cell lines transduced with SLC25A42 shRNA . (**M**) Validation of MESH1 and COASY siRNA knockdown in HT-1080 cells. (**N**) The lowering monomer/dimer ratio of PRDX3 upon erastin treatment were rescued by two independent MESH1 siRNAs. Further knocking down of COASY abolished the protective effect of MESH1 siRNA as determined by Western blots. (**O**) Quantification of the monomer/dimer ratio of PRDX3 upon erastin treatment with or without MESH1 siRNAs or in combination with COASY siRNAs. (**G,K,O**) One-way ANOVA, Tukey's multiple comparisons(**A,B,C,D,E,H**) Two-way ANOVA, Sidak's multiple comparisons. (**A,B,C,D,E,H,I,L,M**) n = 3 independent biological replicates. (**G,K**) n = 5 independent biological replicates. (**O**) n = 4 independent biological replicates. Data represent mean ± SEM.

Supplemental Figure 4

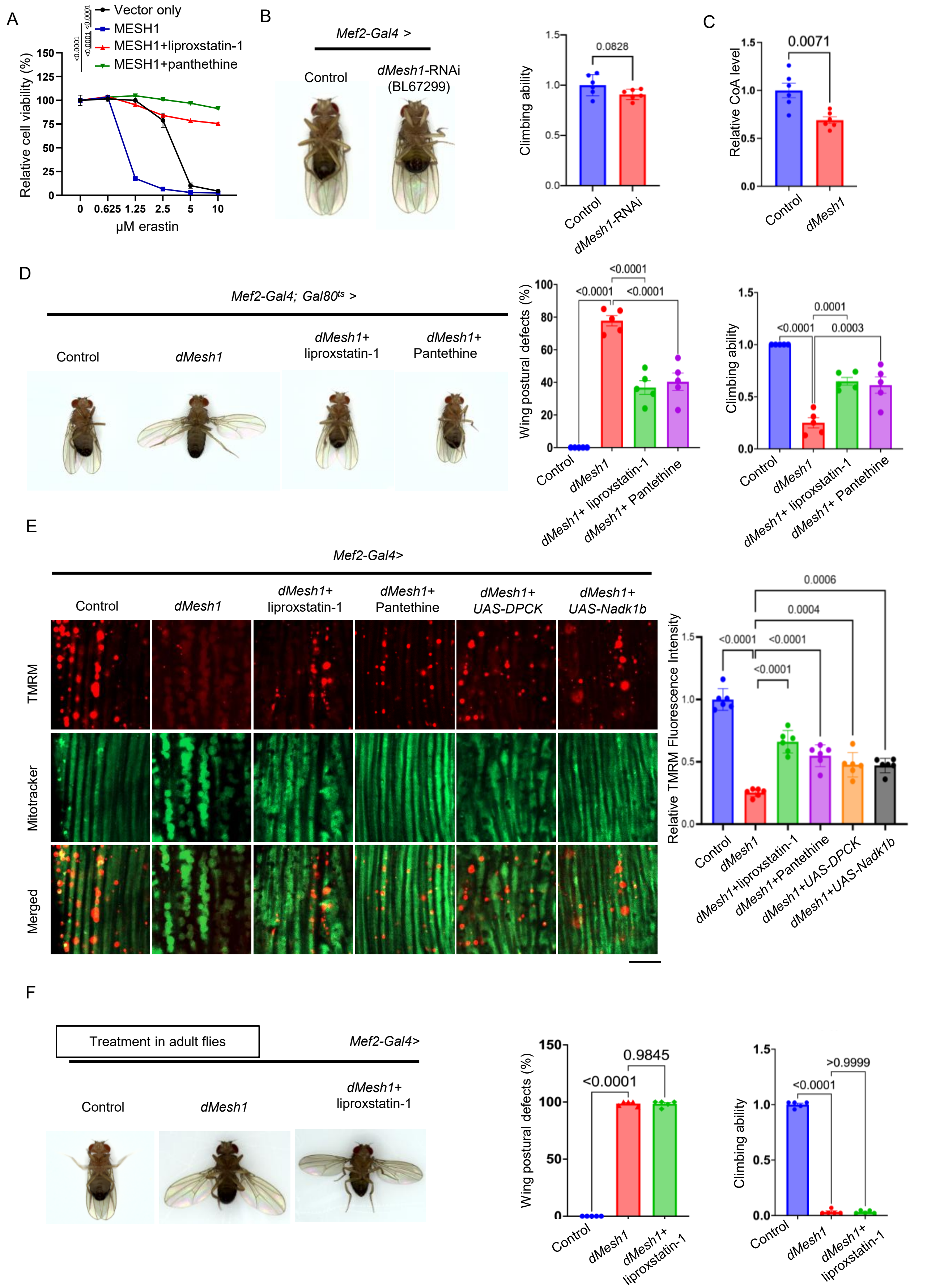

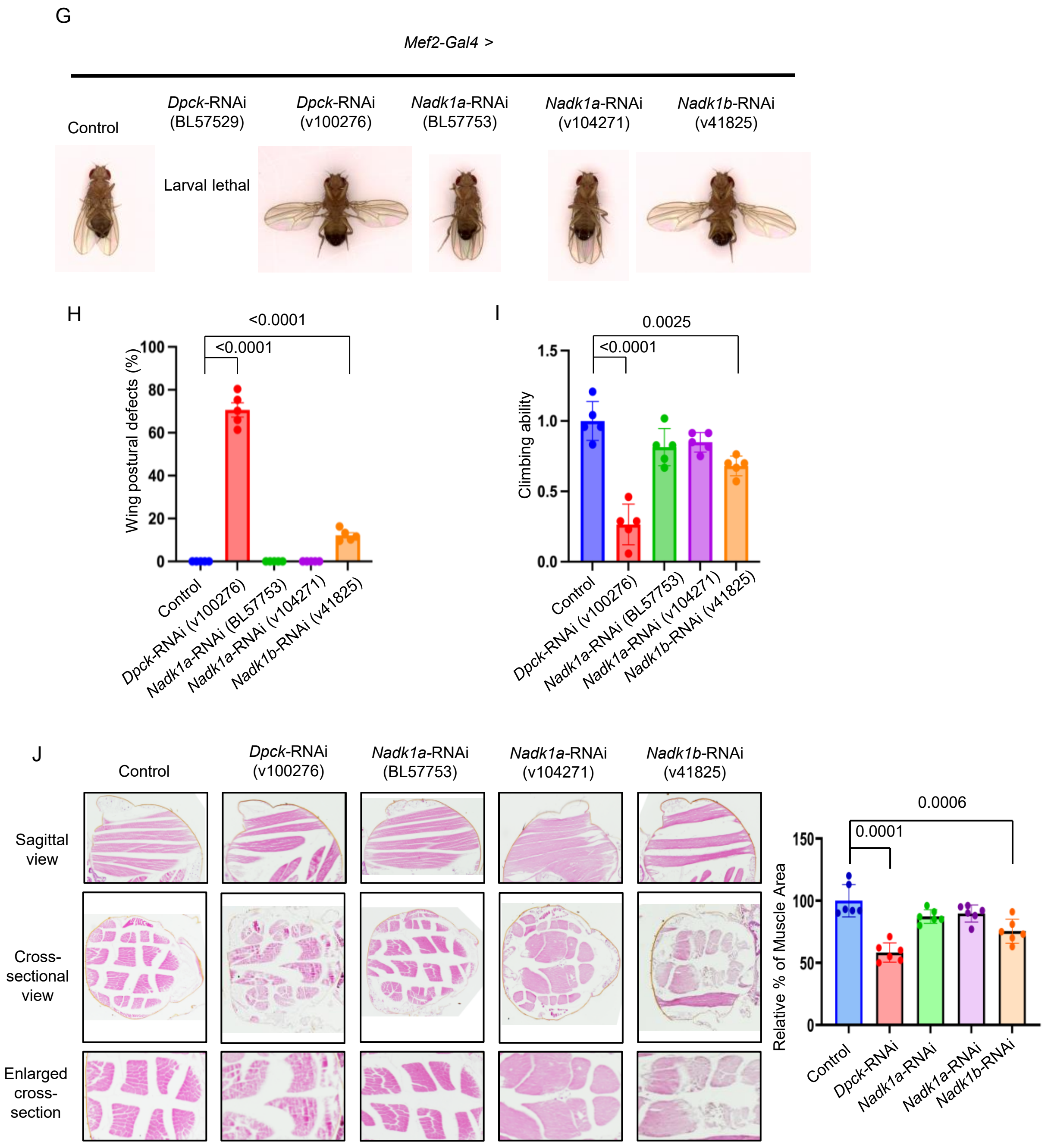

Supplemental Fig.4 Inhibition of CoA or NADPH synthesis leads to muscle atrophy. (**A**) MESH1 over-expression enhanced ferroptosis. HT-1080 cells were transfected with control or MESH1 siRNA for 2 days and erastin treatment alone or with liproxstatin-1 and panthethine for 18 hours for Cell-Titer Glo assay. (**B**) Muscle-specific depletion of *Mesh1* did not produce observable phenotypes in wing morphology or impair muscle function, as assessed by the climbing assay. (**C**) Total CoA levels were decreased in adult fly muscle expressing dMesh1. CoA was quantified from lysates prepared from equal numbers of flies and normalized to total protein. (**D**) Temporal control of *dMesh1* expression using the *Gal80*^{ts} system revealed that adult-specific induction recapitulated abnormal wing posture and climbing defects, which were rescued by Liproxstatin-1 or Pantethine. (**E**) Overexpression of *Mesh1* in skeletal muscles reduced mitochondrial membrane potential, as indicated by TMRM staining. This defect was rescued by Liproxstatin-1 or Pantethine treatment, or by overexpression of *DPCK* or *Nadk1b*. Scale: 10 µm. (**F**) Liproxstatin-1 treatment initiated after phenotype onset failed to reverse these defects. (**F-J**) Depletion of *Dpck* or *Nadk1b*, but not *Nadk1a*, in skeletal muscles resulted in an open wing phenotype (**G-H**), climbing defects (**I**), and muscle shrinkage (**J**). (**D,E,F,H,I,J**) One-way ANOVA, Tukey's multiple comparisons, (**A**) Two-way ANOVA, Sidak's multiple comparisons. (**B,C**) student's t-test, (**A**) n = 3 independent biological replicates, (**D,F,H,I**) n = 5 independent biological replicates, (**B,C,E,J**) n = 6 independent biological replicates, Data represent mean ± SEM.

Parameter	Healthy Controls	Cancer cachexia patients	paired p value
	n=10	n=10	
Age group (distribution)	56.4 ± 17.6	69.5 ± 13.6	0.0809
Gender	4 female, 6 male	7 female, 3 male	NA
Body mass index (BMI) (kg/m^2)	22.56 ± 1.96	18.36 ± 1.71****	<0.0001
Body weight loss (kg)	1.43 ± 0.37	8.58 ± 1.19****	<0.0001
Body weight loss (%)	2.36 ± 0.70	15.94 ± 2.06****	<0.0001
Cancer type	NA	Non small cell lung cancer (3), Liver cancer (5), Metastatic left renal cell carcinoma (1), Gastric adenocarcinoma (1)	NA
Tumor stage	NA	III , IV	NA
CRP (mg/L)	2.21 ± 0.87	23.53 ± 10.81***	0.0001
IL-6 (pg/mL)	0.95 ± 0.52	31.98 ± 19.20***	0.0006
Hb (g/L)	134.2 ± 10.3	97.4 ± 18.3****	<0.0001
Handgrip strength (kg)	29.41 ± 7.61	14.44 ± 5.21***	0.0001

Supplemental Table 1. Demographic and clinical characteristics of study participants. Demographic and clinical characteristics of healthy controls and patients with or without cancer cachexia. Variables include age, sex, body mass index (BMI), weight loss, tumor type and stage, and relevant laboratory parameters. Cancer cachexia was defined as either >5% body weight loss in the past 6 months or >2% weight loss with a BMI <20 kg/m^2. Data are presented as mean ± SD. Statistical significance was assessed by t-test.

Supplemental Table 2. Primer sequences for qPCR.

Gene	Primer orientation	Sequence
MESH1	Forward	GAGCTACACTTTGGGGCACA
MESH1	Reverse	ATGTTCTGACCATCCCTCTGG
ACSL4	Forward	AATACCTGGACTGGGACCGA
ACSL4	Reverse	CCAATCCTGCAGCCATAGGT
COASY	Forward	CCTGGGTCATCGGGCCTAT
COASY	Reverse	GGCCACATAATGTCCGTGAGTAT
NADK	Forward	CACAATGGGCTGGGTGAGAA
NADK	Reverse	TTGGACAGGTAGGAGGAGGG
TXN	Forward	GTGAAGCAGATCGAGAGCAAG
TXN	Reverse	CGTGGCTGAGAAGTCAACTACTA
TXN2	Forward	CTGGTGGCCTGACTGTAACAC
TXN2	Reverse	TGACCACTCGGTCTTGAAAGT
SLC25A42	Forward	TGGTGCCCTTGCCAAAACA
SLC25A42	Reverse	CCCTCGTTGAGGTAGGTGTAG
