# Supplementary material for: MESH1-mediated coenzyme A degradation drives ferroptosis sensitivity and muscle pathology
Source: J Clin Invest. 2026 Apr 25;136(11):e202212. doi: 10.1172/JCI202212 (PMC13221220; doi:10.1172/JCI202212)

Full unedited blots for

MESH1-mediated Coenzyme A degradation drives ferroptosis sensitivity and muscle pathology

Chao-Chieh Lin[1,2*], Joshua Rose[3*], Alexander A Mestre[1,2,3*], Chien-Kuang Cornelia Ding[1,2], Ssu-Yu Chen[1,2], Sze Mun Choy[9], Kah Yong Goh[9], Wen-Xing Lee[9], Yanting Chen[7], Tianai Sun[1,2], Jianli Wu[1,2], Yueqi Chen[8], Yunju Oh[8], Pyeonghwa Jeong[8], Jiyong Hong[6,8], Michael C. Fitzgerald[8], Guo-Fang Zhang[4,5], Hong-Wen Tang[9,10†], Pei Zhou[3†], Jen-Tsan Chi[1,2†]

Corresponding author:

jentsan.chi@duke.edu

peizhou@biochem.duke.edu

hongwen.tang@duke-nus.edu.sg

Full unedited blot/gel for Figure 3E

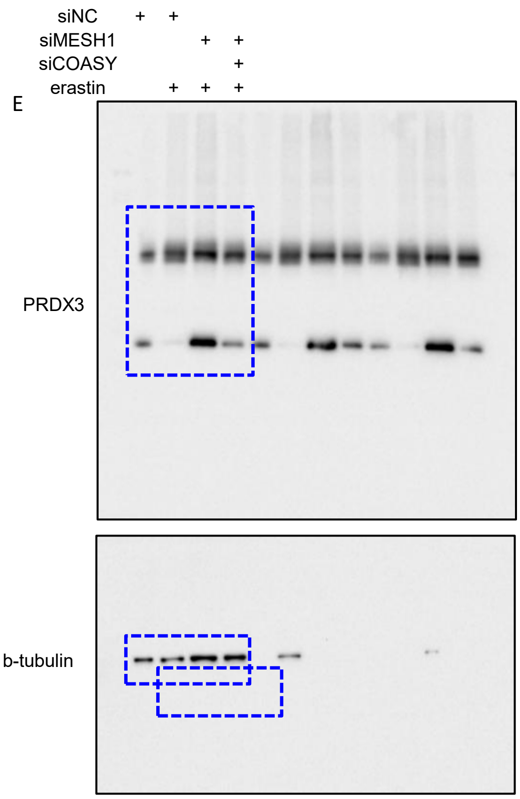

Full unedited blot/gel for Figure 5D

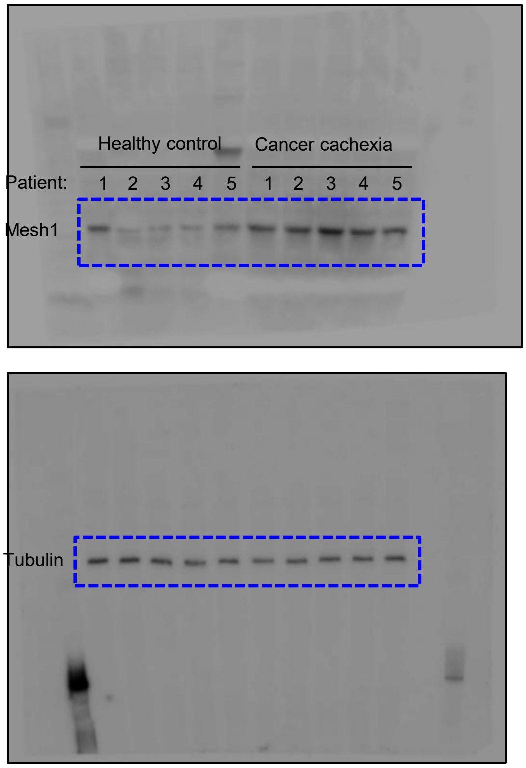

Full unedited blot/gel for Figure S2A

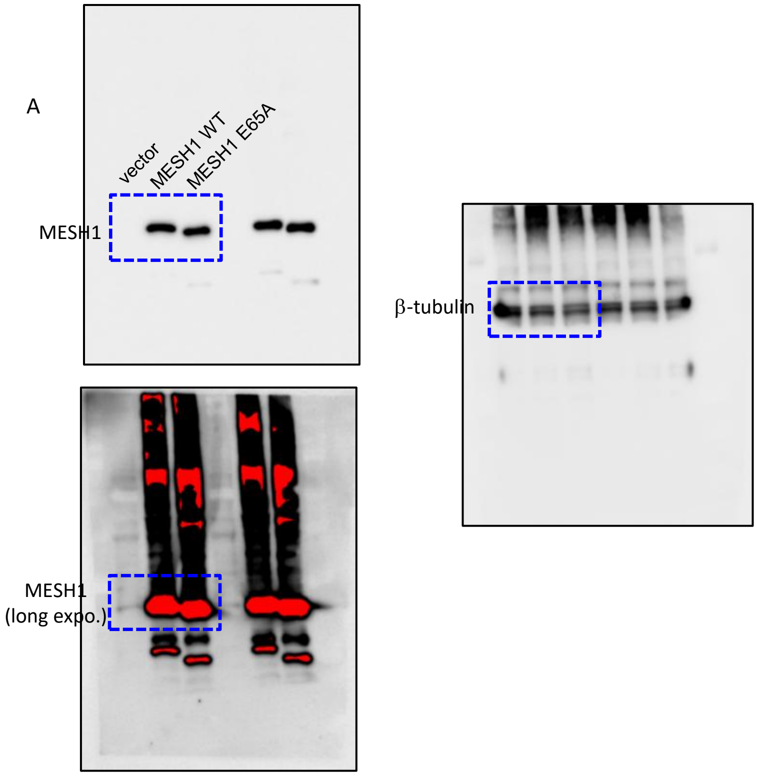

Full unedited blot/gel for Figure S2B

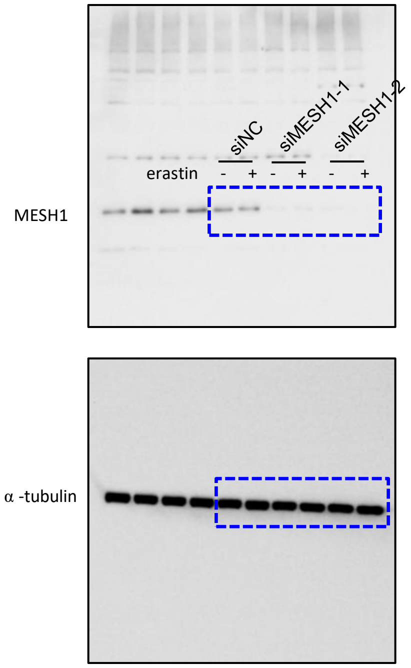

Full unedited blot/gel for Figure S2H

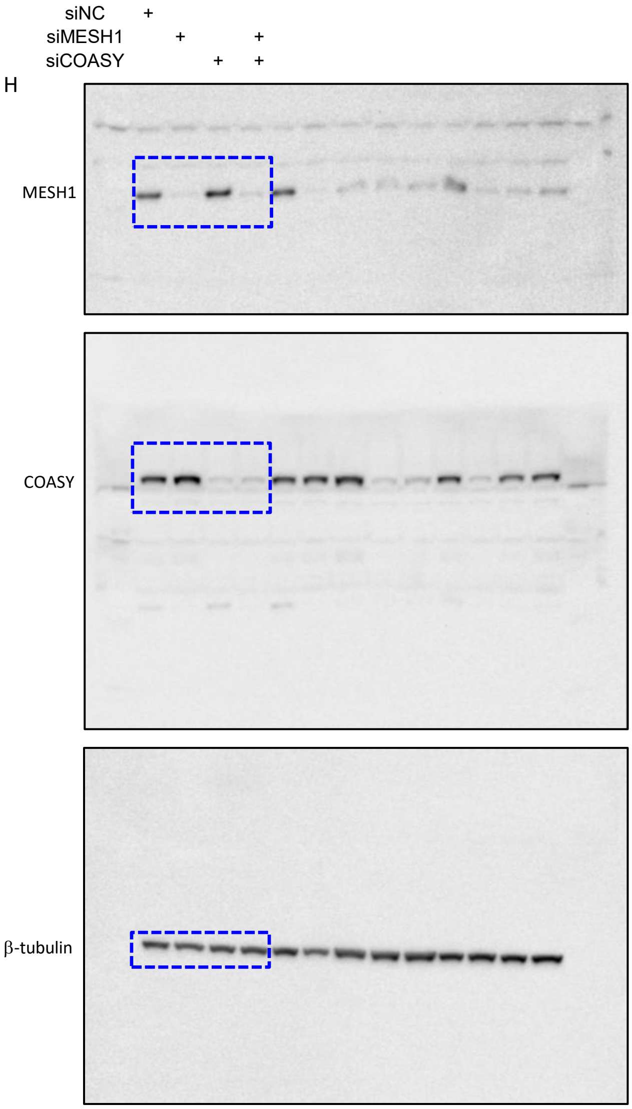

Full unedited blot/gel for Figure S2U

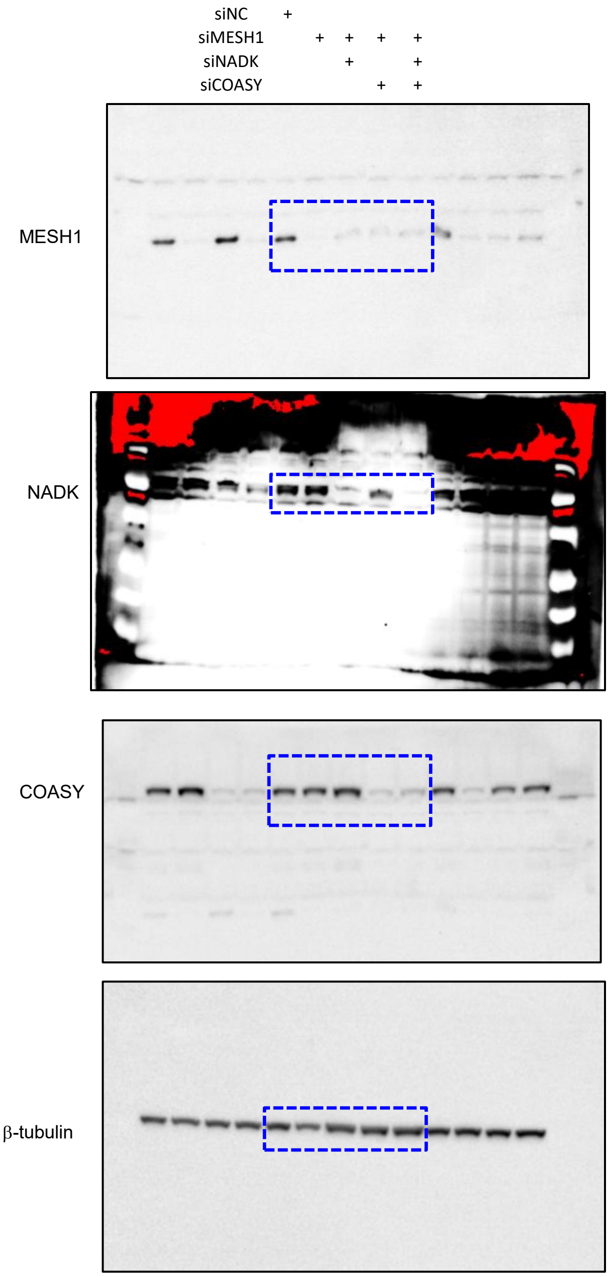

Full unedited blot/gel for Figure S3J

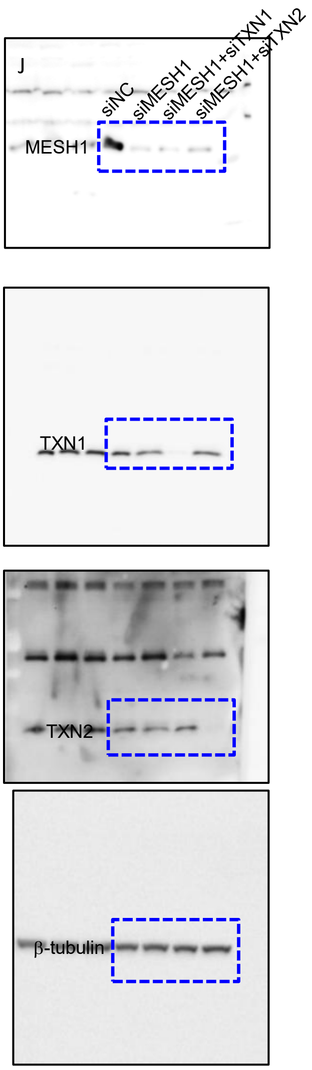

Full unedited blot/gel for Figure S3N

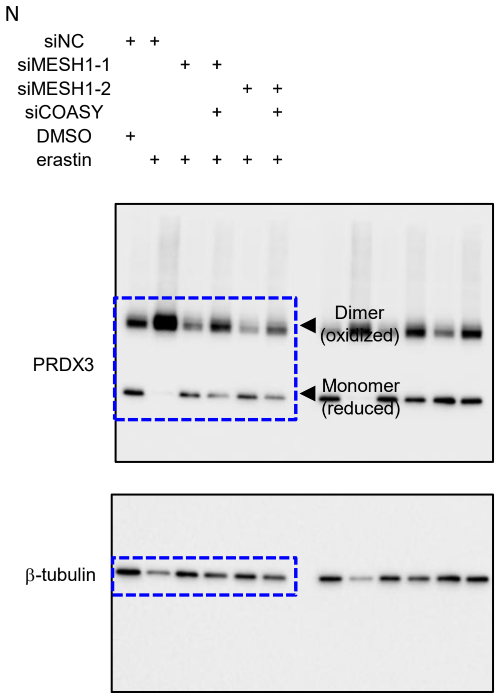

Supplement: Unedited blot and gel images [file jci-136-202212-s061.pdf]
